# Method for automated high performance closed batch cultivation of gas-utilizing methanogens

**DOI:** 10.1186/s13568-025-01872-y

**Published:** 2025-04-29

**Authors:** Walter Hofmann, Marco Orthofer, Nicolás Salas Wallach, Aquilla Ruddyard, Markus Ungerank, Christian Paulik, Simon K.-M. R. Rittmann

**Affiliations:** 1 Archaea Physiology & Biotechnology Group, Department of Functional and Evolutionary Ecology, Universität Wien, Djerassiplatz 1, 1030 Wien, Austria; 2 Acib GmbH, Wien, Austria; 3 Institute for Chemical Technology of Organic Materials, Johannes Kepler Universität Linz, Linz, Austria; 4 Creonia e.U, Perg, Austria

**Keywords:** Microbiology, Biotechnology, Gas fermentation, Microorganisms, Anaerobe, Methanogenic archaea

## Abstract

To advance the utilization of microbial cell factories in gas fermentation processes, their physiological and biotechnological characteristics must be understood. Here, we report on the construction and operation of a novel device, the Gas and Pressure Controller (GPC), which is specifically designed for the automated control of the headspace gas pressure of closed cultivation bottles and facilitates automated gassing, sparging, monitoring and regulation of the headspace volume operated in closed batch cultivation mode in real time.

As proof of concept, the physiological and biotechnological characteristics of four autotrophic, hydrogenotrophic methanogenic archaea were examined to quantify novel physiological limits through the elimination of gas limitation during growth and methane formation. We determined unprecedented high maximum specific methane productivity (qCH_4_) values for: *Methanothermobacter marburgensis* of 169.59 ± 12.52 mmol g^− 1^ h^− 1^, *Methanotorris igneus* of 420.21 ± 89.46 mmol g^− 1^ h^− 1^, *Methanocaldococcus jannaschii* of 364.52 ± 25.50 mmol g^− 1^ h^− 1^ and *Methanocaldococcus villosus* of 356.38 ± 20.79 mmol g^− 1^ h^− 1^. Obtained qCH_4_ of *M. marburgensis* is more than 10-fold higher compared to conventional closed batch cultivation set-ups and as high as the highest reported qCH_4_ value of *M. marburgensis* from fed-batch gas fermentation in stirred tank bioreactors. Furthermore, the GPC demonstrated reliable functionality with *Methanococcus maripaludis*, operating safely and autonomous during long term cultivation. This novel device enables optimal headspace pressure control, providing flexibility in application for various gas-fermenting biotechnological processes. It facilitates near optimal cultivation conditions in semi-continuous closed batch cultivation mode, the analysis of limiting factors in high-throughput experimental design and allows for automated biomass production of autotrophic, hydrogenotrophic methanogens.

**Graphical abstract:**

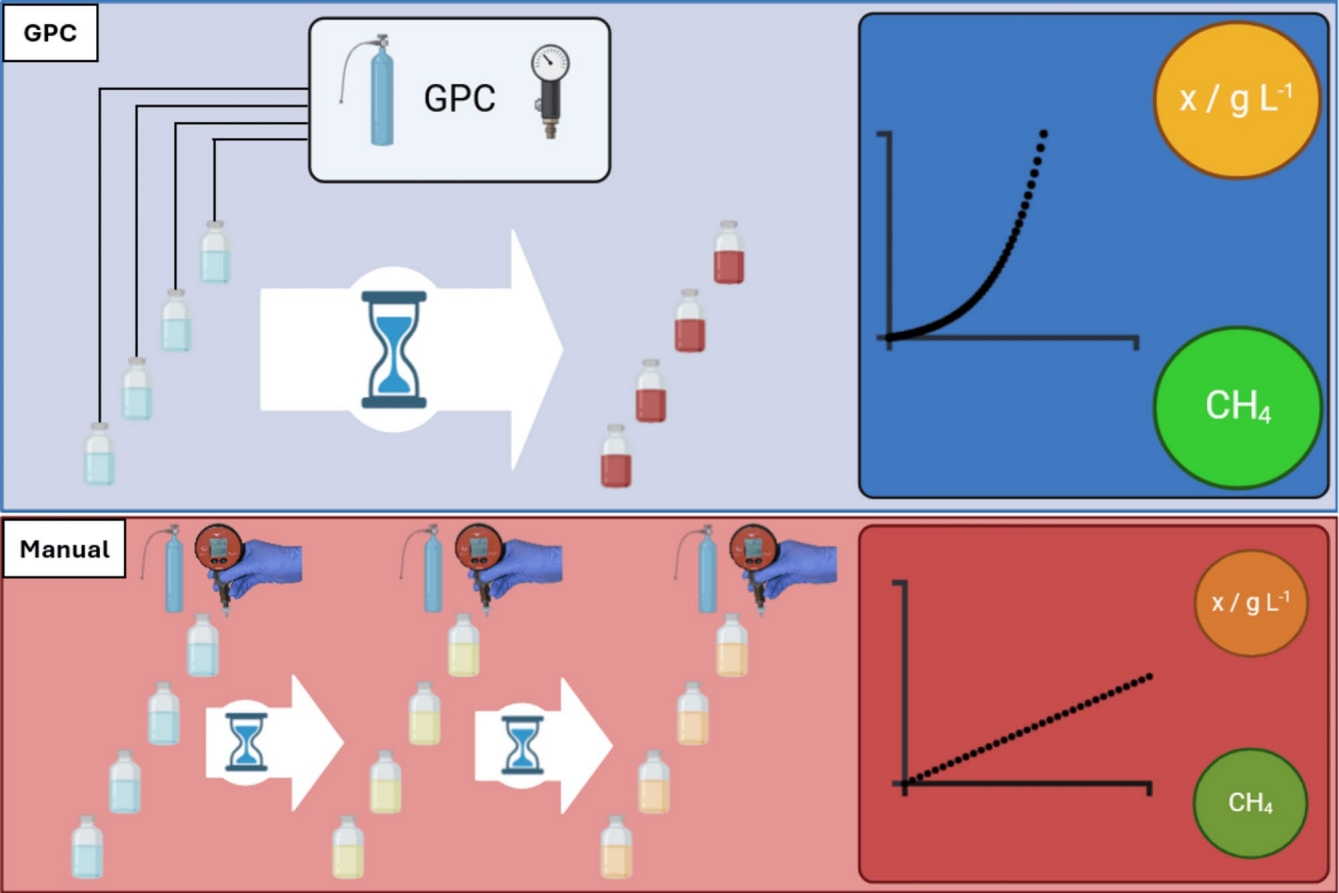

**Supplementary Information:**

The online version contains supplementary material available at 10.1186/s13568-025-01872-y.

## Introduction

In time of limiting resources, microbiology and biotechnology research avenues focus on establishing sustainable production processes. A complete transition towards sustainability may not be solely achieved through one technology but requires a synergy of multiple technologies from different industrial sectors and advances from various scientific fields. Two promising and rapidly emerging biotechnological fields are gas fermentation (Liew et al. 2022; Woern and Grossmann 2023) and archaea biotechnology (Straub et al. 2018; Pfeifer et al. 2021). Archaea are a group of prokaryotic microorganisms that exhibit unique biochemical, metabolic and physiological properties that allow application in a wide variety of industrial applications (Pfeifer et al. 2021; Carr and Buan 2022; Aparici-Carratalá et al. 2023). Despite their physiological and biotechnological potential, archaea remain largely underexplored in both scientific research and technological applications. However, many different compounds can be nowadays produced by archaea (Pfeifer et al. 2021; Rittmann et al. 2021) and major advancement in archaea biotechnology have been recently achieved (Rittmann et al. 2023a, b, c, d), with a few archaeal production processes currently being scaled-up beyond lab-scale (Pfeifer et al. 2021). Significant improvements using archaea have also been made in the context of gas fermentation processes, converting gaseous C1 compounds into gaseous or liquid compounds. In this regard, biohydrogen production through the water gas shift reaction (Kim et al. 2013, 2017; Rittmann et al. 2015b) as well as the conversion of carbon dioxide (CO_2_) and molecular hydrogen (H_2_) into methane (CH_4_) (Seifert et al. 2014; Rittmann et al. 2018; Mauerhofer et al. 2021) have been thoroughly studied. These bioprocesses may act as carbon utilization avenues, reducing global carbon emissions by converting inorganic carbon into organic material through autotrophic organisms.

Methanogenic archaea (methanogens) (Lyu et al. 2018, 2022) are anaerobic organisms and possesses ecological flexibility that allows them to inhabit various anoxic environments including the gastrointestinal tract (Borrel et al. 2023) and could possibly thrive in extraterrestrial environments (Taubner et al. 2018). They utilize a wide range of different substrate types including methyl compounds, acetate and C1 compounds (Kurth et al. 2020). Autotrophic, hydrogenotrophic methanogens are archaeal cell factories for H_2_/CO_2_ to CH_4_ conversion (Rittmann et al. 2014, 2018; Rittmann 2015) and may be employed for proteinogenic amino acid production (Rittmann et al. 2023a, b, c, d; Taubner et al. 2023; Reischl et al. 2025). However, a major obstacle that remains is to characterize methanogens in high-throughput screening settings– before bioprocess development shall be initiated. Examining the physiological boundaries of methanogens under comparable and highly reproducible cultivation conditions is therefore essential for identifying and prioritizing strains for their subsequent optimization. Several studies already emphasize the importance of bioprocess factors for regulating the physiological output of methanogens, including gassing rates, pH, stirring rates, temperature, media composition and dilution rates in various cultivation settings (Seifert et al. 2014; Rittmann et al. 2015a, 2018; Pappenreiter et al. 2019; Mauerhofer et al. 2021).

Anaerobic cultivation of gas-utilizing microorganisms must meet specific requirements, including the prevention of molecular oxygen (O_2_) exposure, as O_2_ is toxic to varying degrees for the strict anaerobic organisms (Mauerhofer et al. 2019; Hanišáková et al. 2022). The most used cultivation technique in anaerobic microbiology and biotechnology laboratories is closed batch, commonly performed in serum bottles (Taubner and Rittmann 2016; Mauerhofer et al. 2019, 2021; Hanišáková et al. 2022) and, in the case of autotrophic, hydrogenotrophic methanogens, under slight overpressure conditions (Taubner and Rittmann 2016). Later, scale-up of bioprocesses occurs in bioreactors, during which continuous supply of gaseous substrates in fed-batch (Abdel Azim et al. 2017) or continuous culture mode (both liquid and gas feed) is employed (Seifert et al. 2014). The bottleneck in closed batch, but also in bioreactors, is (usually) not the catalysis rate of the biomass, but the gas transfer from the gas into the liquid phase. It is therefore necessary to optimize the gas transfer rate (GTR) using biochemical bioengineering methods (Rittmann et al. 2015a; Takors et al. 2018). This can be achieved by increasing the partial pressure of the respective gas, which is why gas fermentations are specifically carried out under elevated pressure (Takors et al. 2018; Taubner et al. 2018; Pappenreiter et al. 2019; Mauerhofer et al. 2021), or though increasing the gas-liquid mass transfer coefficient (k_L_a) (Takors et al. 2018). Cultivation in closed batch cultivation mode is usually performed before process development is initiated or for obtaining seeding cultures for inoculation (Rittmann et al. 2015a). This is due to the cost-effectiveness, low expertise requirements of operators and suitability for high-throughput experiments compared to operating bioreactors. Successful outcomes of closed batch cultivations include the assessment of physiological parameters by investigating the methane evolution rates of autotrophic, hydrogenotrophic methanogens (Mauerhofer et al. 2021), studies of analysis of growth behavior under elevated heavy metals and volatile fatty acids concentration (Abdel Azim et al. 2018) as well as media optimization studies (Abdel Azim et al. 2017; Mauerhofer et al. 2021). Furthermore, closed batch cultivations are an integral part of biomass production pipelines (Palabikyan et al. 2022). However, during closed batch mode, sub-optimal supply of the gaseous substrate prohibits balanced growth shifting the growth kinetics to a disproportionate pattern, labelling it unsuitable for modelling and scale up of biomethanation processes. Therefore, optimizing cultivation conditions in closed batch setups is crucial to be able to draw significant conclusions in screening experiments, even in a small-scale high-throughput design.

In this article, we present the functionality and operation of an innovative device, designed for automated gassing, purging, sparging and pressurizing of sealed microbial cultivation bottles independent of the process volume for closed batch cultivations– the Gas and Pressure Controller (GPC). This novel apparatus allows for continuous real-time pressure monitoring throughout the cultivation process. In this proof-of-concept study, convenient quantification of gas conversion and CH_4_ production kinetics of five autotrophic, hydrogenotrophic methanogens is demonstrated. The utilization of the GPC facilitates the application of a specific stoichiometry during gas conversion according to the metabolic requirements of the organisms, which leads to a noticeable pressure drop during gas fermentation. The GPC overcomes gas limitations by constant and automated adaptation of the headspace gas pressure, therefore allowing optimal growth in unimpeded conditions. This combination of the advantages of closed batch gas fermentation under gas-unlimited conditions was previously not possible and represents a major leap forward in closed batch cultivation techniques.

## Materials and methods

### Strains and media

For the proof-of-concept demonstration, five autotrophic, hydrogenotrophic methanogens *Methanothermobacter marburgensis* DSM 2133, *Methanotorris igneus* DSM 5666, *Methanocaldococcus jannaschii* DSM 2661, *Methanocaldococcus villosus* DSM 22612 and *Methanococcus maripaludis* S0001 were cultivated in closed batch utilizing the GPC. *M. maripaludis* S0001 is a mutated strain and originates from the wild-type strain S2. It was generated by deleting the gene encoding for the hypoxanthine phosphoribosyltransferase (MMP0145) and introducing the rep gene from the *Methanococcus* shuttle vector pURB500 (Walters et al. 2011; Long et al. 2021). The cultivation medium ‘282c_18’ was used for *M. villosus*, while ‘282c_30’ was applied for both *M. jannaschii* and *M. igneus*. Exact media compositions were derived from a previous study (Mauerhofer et al. 2021), with a single modification of omitting NaHCO_3_. The defined medium for *M. marburgensis* was prepared identical to the previously published recipe (Abdel Azim et al. 2018). The cultivation media for *M. maripaludis* was applied as previously described (Palabikyan et al. 2022). However, sodium acetate was excluded from the recipe, as the metabolism of *M. maripaludis* was shifted to CO_2_ as sole carbon source. All methanogens were cultivated according to their optimal pH and temperature. The pH was adjusted aerobically before complementing the media with Na_2_S and L-Cysteine. To demonstrate pH changes under anaerobic, CO_2_ saturated conditions with elevated pressure and following the addition of alkaline solution Na_2_S, the defined media (MM) was pressurized to 15 bar absolute and analyzed at 65 °C and room temperature (RT). These experiments were conducted using the simultaneous bioreactor system (SBRS-II) (Orthofer *et al*., submitted for publication). The cultures were supplied with a premixed gas (20 Vol.-% CO_2_ in H_2_) (Air Liquide GmbH, Schwechat, Austria) as gaseous substrate and was introduced manually through an offline gassing manifold (pre-culture) or automated with the GPC.

### Closed batch cultivation—pre-cultures

The pre-cultures of the four thermophilic/hyperthermophilic methanogens were cultivated in pressure-resistant 500 mL Schott bottles (DURAN^®^ pressure plus + GL 45; DWK Life Sciences GmbH, Wertheim, Germany) using established closed batch system technique, adapted for larger culture volumes of 200 mL (Taubner and Rittmann 2016). The anaerobic bottles were sealed with butyl rubber stoppers and fastened with perforated caps (Ochs Laborbedarf, Bovenden, Germany). The pre-culture of *M. maripaludis* was initially cultivated in serum bottles before performing GPC experiments as previously established (Taubner and Rittmann 2016). All bottles were anaerobized by a total of ten repetitions of gassing and subsequent vacuuming to 0.5 bar with H_2_/CO_2_ (4:1) prior to inoculation. Incubation of pre-cultures were performed by pressurizing to 1.5 bar for Schott bottles and 3 bar overpressure for serum bottles. Sterile conditions were ensured by connecting a 0.20 μm cellulose acetate filter (9055511; LLG Labware, Meckenheim, Germany) while gassing and pressure measurements with a digital manometer (303005.0003; Keller, Winterthur, Switzerland). Growth was observed by measuring the optical density (OD) with a Specord 200 Plus spectrophotometer (823-0200P2; Analytik Jena, Jena, Germany) at 578 nm. The thermophilic/hyperthermophilic hydrogenotrophic methanogens were incubated using a GFL 1083 shaking water bath (Gesellschaft für Labortechnik mbH, Burgwedel, Germany) filled with heating bath fluid ROTITHERM^®^ (Carl Roth GmbH + Co. KG, Karlsruhe, Germany). The internal temperature was maintained according to the specific temperature requirements. Set optimal temperatures were 65 °C for *M. marburgensis*, 88 °C for *M. igneus*, 80 °C for *M. villous* and 85 °C for *M. jannaschii* with a constant unidirectional agitation of 100 rpm during the pre-culture phase. *M. maripaludis* was cultivated in a Premium Double Layer Shaking Incubator 170 L ZWYR-2102 C (LABWIT Scientific, Burwood East, VIC, 3151) at 37 °C and 180 rpm orbital shaking. An overview of the cultivation conditions is shown in Table 1.

### Determination of dry biomass weight

To analyze the specific CH_4_ production rate (qCH_4_) of the methanogen, a conversion factor (x) for optical density to dry biomass was determined for investigated methanogen except for *M. maripaludis*. The cultures were prepared as described above. Filters (Whatman^®^ regenerated cellulose RC58 filter discs 0.2 μm, Cytiva, Massachusetts, US) were dried overnight at 105 °C and weighed the following day. The OD_578_ of the cell suspension was measured, the diluted cultures filtered (10 mL) in triplicates and subsequently washed three times with the corresponding medium. The filters were subsequently dried overnight at 105 °C and the total weight of the filters measured. Detected dry biomass can be subsequently converted to the initial OD_578_.

## GPC

### Application and set-up of the GPC

One GPC unit consists of four individual channels and allows for the supply and exhaust of gas in four pressurized cultivation vessels (Figs. S1-S3). Four external gas connections are available and can be used to supply different gases to the culture. The gas flow is regulated by 8 Solenoid valves (SV) (SV1-8, Figs. S1 and S2) (1913CA302; Ningbo Brando Hardware Co., Ltd., Zhejiang, China) placed in two parallel rows. Molecular nitrogen (N_2_) supply (Fig. S2, valve position #8) (not used) and a 4:1 H_2_/CO_2_ gas bottle (Fig. S2, valve position #7) were connected while one open position (Fig. S2, valve position #5) functioning as an off-gas discharge to ventilate the headspace volume. The inlet pressure was adjusted using the pressure regulators situated in the lab. Additional downstream throttle valves were integrated for better fine tuning (Fig. S1, NV1-4; Fig. S2). Opening two valves in parallel directs the gas flow from the high-pressure laboratory gas lines (~ 5 bar) to the lower-pressure cultivation bottles. If pressure needs to be released from the cultivation bottles, the gas is vented through an exhaust valve (valve position #5). A total of four distinct and autonomously executable sequence programs in CSV format can be generated and uploaded to the controller. Real-time pressure changes are monitored using manometers (Fig. S1, PIC 1–4; Fig. S2, M1-4) (RL-131-II-1 A; Anhui Ruiling Meter Manufacturing Co. Ltd, Anhui, China) respectively. All elements are interconnected with pressure resistant tubing (Master-Tube PUR 98 A– Masterflex, Gelsenkirchen, Germany). The GPC was fitted in a fume hood to facilitate a rapid extraction of released gases. Two additional devices are placed outside and contain power supply, system control and facilitate data monitoring and recording. In order to maintain anaerobic conditions inside the serum bottles, a T-Fitting connector is used to join the valve and manometer tubing, with the remaining position connected to a pressure-resistant bottle using a Luer-Lock adapter (male) with a sterile needle. PTFE gas tape is applied to all connections (Manometer, T Fitting, needle) to ensure tight sealing of the system. The cultivation bottles were placed in the corresponding incubator. The same incubators were employed as previously described. For the mesophilic strains, the gas lines were conducted through an opening on the side of the incubator into its interior and connected to the cultivation bottles. During mesophilic cultivation, a sterile filter was installed directly after the valves (Fig. S2, SV5-8) to increase the sterility of the system.

**Fig. 1:**
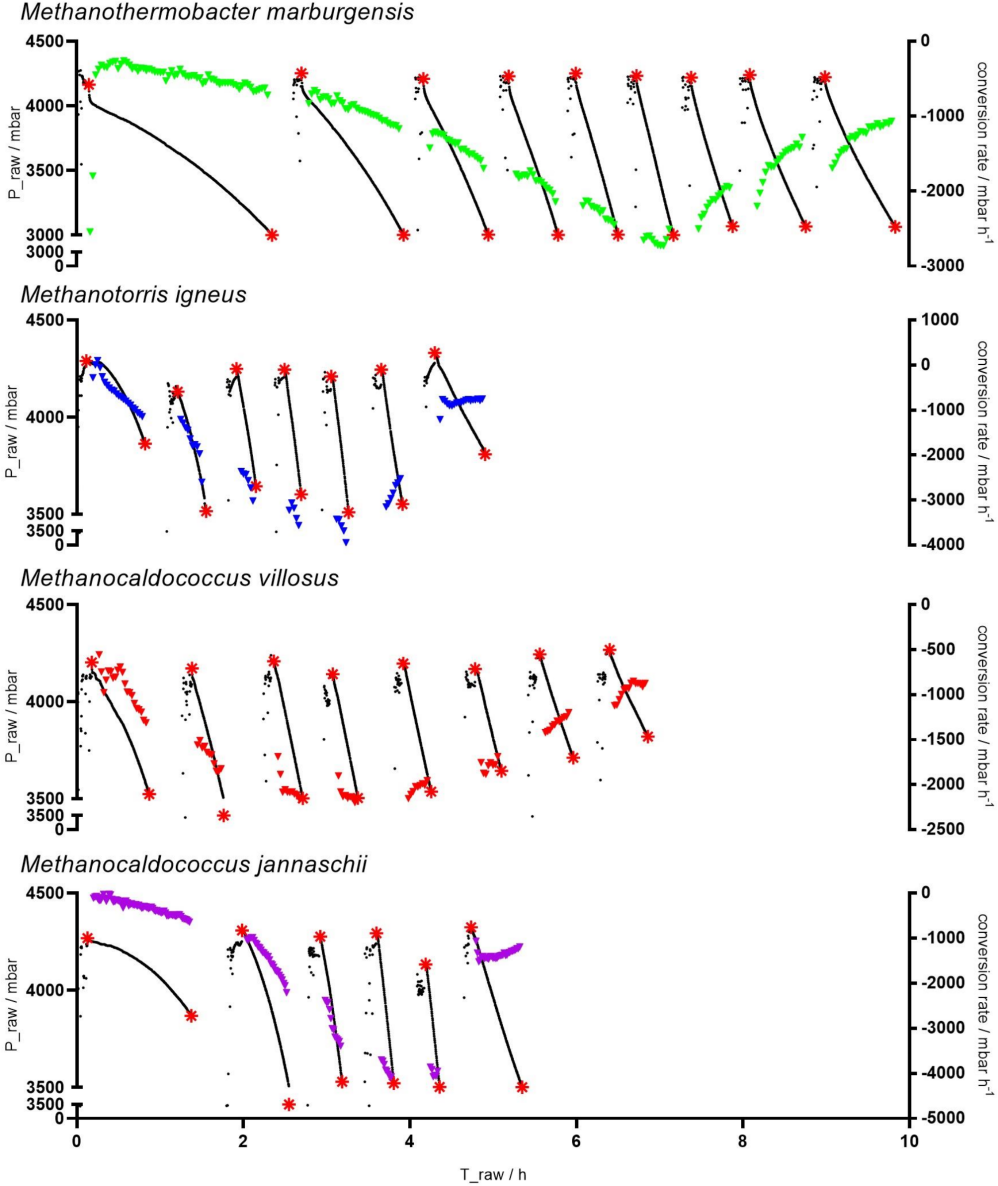
Overview of the pressure kinetics of the thermophilic/hyperthermophilic autotrophic and hydrogenotrophic methanogens. Black dots indicate raw data (prior to trimming and smoothing) of the first replicate. Start and end of the cultivation time is highlighted by red stars. The conversion rates / mbar h^−1^ are shown in color

**Fig. 2:**
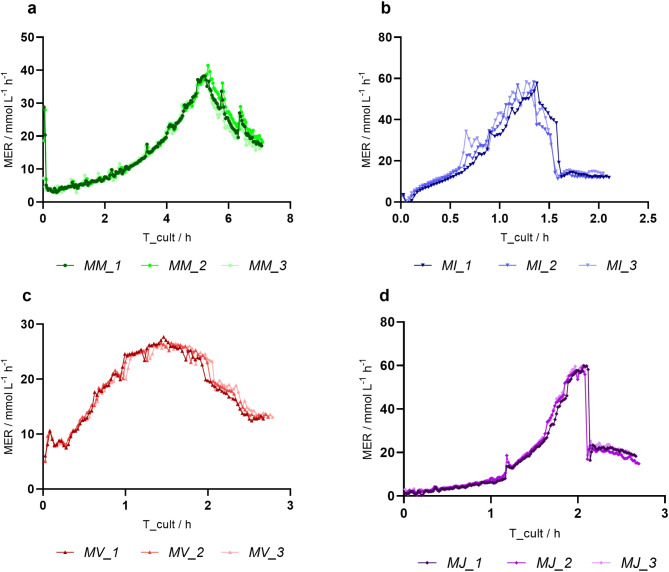
MER / mmol L^−1^ h^−1^ determined over the cultivation time for **a*** M. marburgensis (MM)*, **b*** M. igneus (MI)*, **c*** M. villosus (MV)* and **d*** M. jannaschii (MJ)*. All experiments are n = 3. Individual experiments are shown

**Fig. 3:**
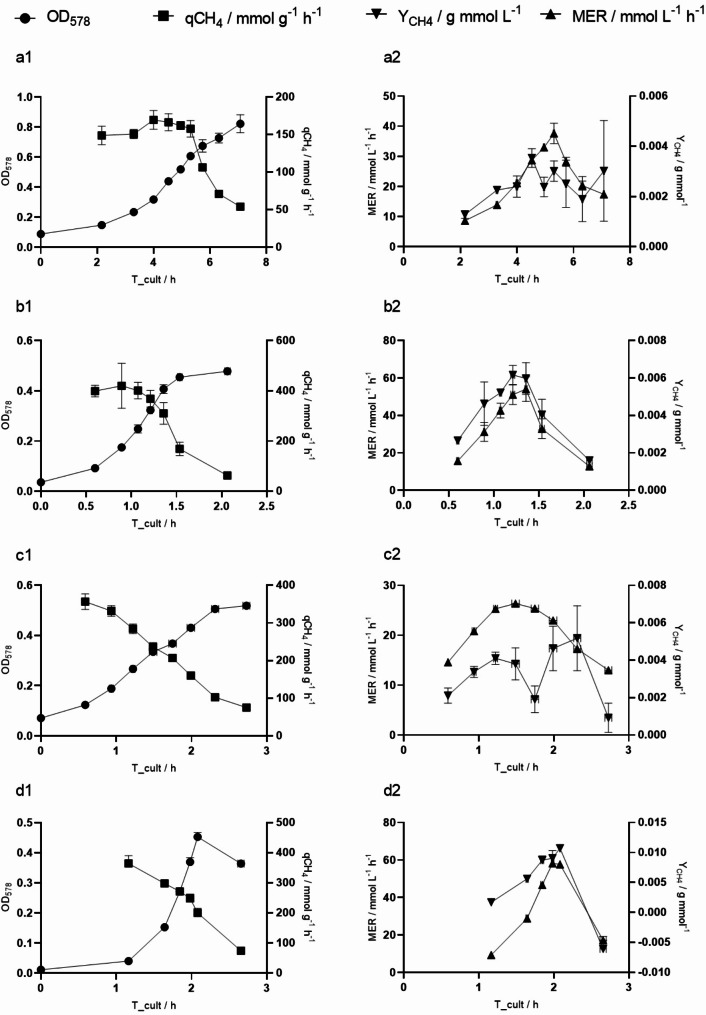
Overview of OD578, MER/mmol L^−1^ h^−1^, qCH_4_/ mmol g^−1^ h^−1^ and YCH_4_ g mmol^−1^ strain specific values plotted over cultivation time. Shown results from **a** (1-2) *M. marburgensis*, **b** (1-2) *M. igneus*, **c** (1-2) *M. villosus* and **d** (1-2) *M. jannaschii* represent measured values at the end of each cycle. Horizontal error bars present due to slight differences in the cycle length

**Fig. 4:**
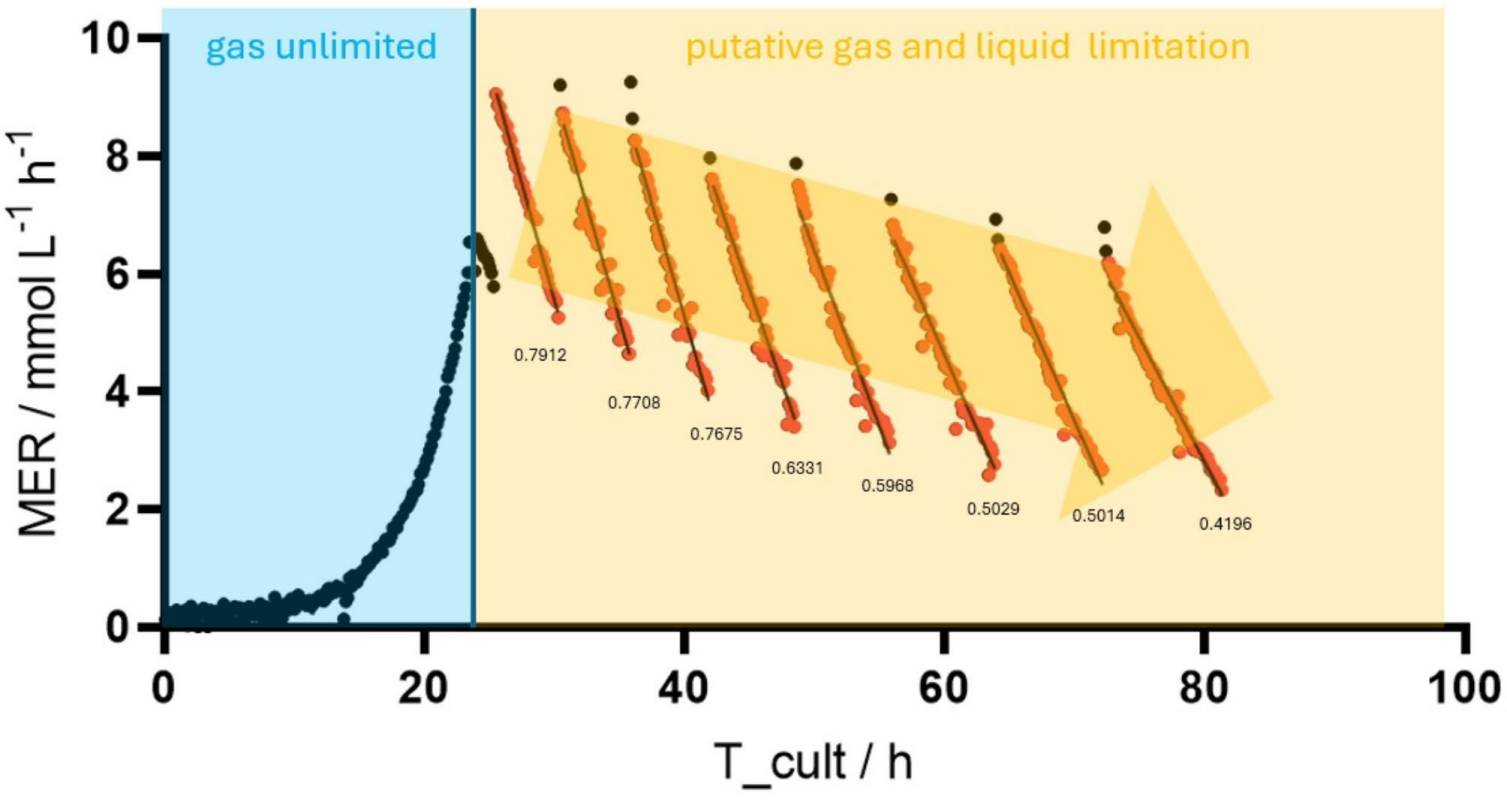
MER/mmol L^−1^ h^−1^ determined over an extended cultivation time (86 h) for *M. maripaludis*. Phases of putative gas and liquid limitation are indicated. Slopes were calculated from linear regression, Only the first experiment is shown. Experiment was performed in triplicates (n = 3). Additional experiments can be found in the supplementary materials (Fig. S13)

### Standard operating procedure of the GPC

A precise gassing layout must be defined before the start of cultivation and uploaded to the controller as a CSV file via a micro-SD card. Two sets of experiments were conducted to illustrate the functionality of the system. To assess physiological parameters of *M. marburgensis*,* M. igenus*,* M. villous* and *M. jannaschii* the GPC was paused after each methanation cycle and the optical density (OD_578_) measured. The unidirectional agitation rate was set to 250 rpm to facilitate maximum gas entry into the liquid phase. The cultivation of *M. maripaludis* was performed over several days without biomass detection to facilitate unsupervised cultivation at orbital shaking of 180 rpm. Therefore, the methanation phase was looped with the flushing of the cultivation bottles (20x) to guarantee automated gas supply during the cultivation. Prior to the methanation process and during sampling steps, the headspace volume of the culture vessel is exchanged by pressurizing with H_2_/CO_2_ gas mixture followed by subsequent release (Flushing phase). At the start of each methanation phase, a pressure of 4 bar absolute is applied for cultivation in serum bottles and 2.5 bar for Schott bottles. The gas consumption, determined as the absolute pressure drop, was set to 1 bar cycle^− 1^ in the case of *M. marburgensis* and *M. maripaludis* and 0.5 bar cycle^− 1^ for the hyperthermophilic organism in order to increase the total amount of gassing cycles. An overview and detailed description of used programs can be found in the supplementary materials (Fig. S4).

The following protocol delineates growth of autotrophic, hydrogenotrophic methanogens for biomass production and quantitatively assessment of their methanation. Unless specified otherwise, all preparatory procedures were performed in an anaerobic glove box (Vinyl Anaerobic Chambers; Coy Laboratory Products Inc, Michigan, USA). A single controller was utilized to monitor and supply gas to three individual cultures simultaneously (*n* = 3), while an additional negative control was cultivated (not shown).

1. Inoculation.
  a Harvest the pre-cultures in active growth phase by centrifugation at 5,000 g for 15 min (RT).
  b Discard the supernatant and re-suspend the pellet in fresh medium (OD_578 ~_ 0.05).
  c Transfer 30 mL of re-suspended culture into a 60 mL serum bottle and seal as previously described.

Note *M. maripaludis* is known to be a fragile organism due to its thin S-layer (Jones et al. 1983; Palabikyan et al. 2022), therefore centrifugation was avoided during the inoculation process and was performed with a sterile needle and syringe. *M. maripaludis* was cultivated in 1 L pressure resistant Schott bottles with a total of 204.175 mL culture volume (2 Vol.-% inoculum).

2 Start cultivation.
  a Place culture vessel into pre-heated incubator and wait until process temperature is reached.
  b Switch on the GPC.
  c Attach a sterile needle to the tubing (luer lock).
  d Start the program and wait until air is flushed out of the tubing, then penetrate the septum with the needle.
  e Initiate methanation phase by start shaking of the incubator after target pressure is reached.

3 Sampling (optional).
  a After the automatic stop of the gassing station, the injection needle can be pulled out carefully from the septum.
  b Remove the serum bottle from the incubator and take a sample (0.7 mL) with a sterile syringe and needle for measuring optical density.
  c Reinsert the bottle and repeat steps 2 d-e. to restart the methanation phase.

4 End experiment.

After completing the experiment, the agitation can be stopped and the needle removed from the cultivation bottle.

### Data analysis

An algorithm was developed for fast and easy processing of the raw data in a singular step and was subsequently executed on Matlab 2021b (MathWorks; Natick, Massachusetts, USA). It allows for trimming and smoothing of the raw data as well as the calculation of the most important physiological parameters. A sampling rate of 10 s was directly configured within the GPC software to minimize the initial amount of data points.

The following filters were applied to facilitate accurate data trimming and were executed sequentially.

i Elimination of data points below a specified pressure threshold (*min-Pressure*). The experiment stops when a certain pressure threshold is reached. Therefore, data points below this limit do not represent the actual pressure evolution.
ii Organization of data into cycles. A cycle is defined from the maximum pressure to a defined threshold or minimum recorded pressure. Data that does not meet this definition is eliminated.
iii Elimination of short-duration cycles (*len_cycles*): Due to the differentiated re-pressurization of the cultures, the pressure gauges are affected by the opening and closing of valves, resulting in small cycles that are removed.
iv Elimination of cycles with no activity (*pressure_drop*). Cycles devoid of a minimum pressure drop of 50 mbar imply no catalytic activity and are removed.
v Elimination of data points that are not consecutive within a cycle. It is anticipated that the data within each cycle progresses sequentially and, at most, deviates by a factor of 2 times the measurement time.

A preset of variables can be manually adjusted to allow for individual evaluation of a wide range of different pressure kinetics. This involves the *move_max* function, which erases a specific number of data points following the peak pressure point after the initial trimming steps. The *calculation* value determines the number of data points used for calculation, while the *average* function adjusts the smoothing factor. CH_4_ production was quantified based on the stoichiometry of the biomethanation reaction from H_2_/CO_2_ as previously described.(Taubner and Rittmann 2016). This calculation is based on the pressure drop concomitant to the methanation, converting five mol of gaseous substrate to 1 mol of CH_4_ (I, II).

I $$\:{p}_{CH4}=\frac{{p}_{before}-{p}_{after}}{4}\left[Pa\right]$$

II $$\:MER=\frac{\varDelta\:{n}_{CH4}}{\varDelta\:t*V}\left[mmol{L}^{-1}{h}^{-1}\right]$$

As it is assumed that only around 5% of the carbon flux is directed into the biomass, the biomass production was omitted for the mass balance and full conversion of CO_2_ into CH_4_ assumed (Schill et al. 2000). The volumetric methane evolution rate (MER / mmol L^− 1^ h^− 1^), the conversion rate / mbar h^− 1^ and the produced mol of CH_4_ (n_CH4_ / mol) are calculated over the whole cultivation time automatically with the algorithm. Additionally, the specific CH_4_ production rate (qCH_4_ / mmol g^− 1^ L^− 1^), biomass concentration (dry cell weight) (x / g L^− 1^), biomass production rate (r_x_ / g L^− 1^ h^− 1^) and the growth yield (Y_CH4_ / g mmol^− 1^) can additionally be calculated. Calculations of qCH_4_ are based on the final MER values of each cycle, coinciding with the time point at which growth was monitored. The results are plotted against the cultivation time (T_cult / h) which represents the duration of the experiment without accounting for the time intervals between the methanation cycles. Two different versions of the algorithm are provided. The standard version assumes biomass data is available, while in the second version (v2), biomass-specific values are calculated only if a biomass file is present. The culture volume (V_culture_ / mL), the total gas volume in the headspace (V_headspace_ / mL), and possibly the OD_578_ to dry biomass coefficient (x) were additionally supplied. A reduction in culture volume and an increase in headspace volume of 0.7 mL resulting from sampling were considered after each cycle. An overview of all parameters can be found in Table 1. Definition of the cultivation time and calculation of the specific growth rate (exponential formulation) (µ / h^− 1^) were performed separately.

The homogeneity of variance was tested in between cycles with a Levene’s test. Statistical relevance was verified further with an ANOVA and subsequent Post hoc analysis (only performed for the thermophilic/hyperthermophilic organisms). Only common cycles before reaching the inflection point were examined.

**Table 1:** Overview of all cultivation parameters and variables specific to each organism.

Strain	T / °C	pH	V_culture_ / mL	V_headspace_ / mL	Δpressure / bar cycle^− 1^	x	Calculation	Average	Move_max
*M. marburgensis*	65	7	30	38.228	1	0.393	12	9	10
*M. igneus*	88	5.7	30	44.889	0.5	0.429	10	9	1
*M. villosus*	80	6.5	30	38.228	0.5	0.334	10	9	5
*M. jannaschii*	85	6	30	44.889	0.5	0.635	6	5	10
*M. maripaludis*	37	6.9	204.175	1006.025	1	-	50	30	30

Volumes displayed refer to cultivation with the GPC. V_headspace_ / mL is the total volume of gas from both the bottle headspace and the tubing. Variables for fine-tuned calculation of physiological parameters are given in timepoints (10 s)

## Results

Measured OD_578_ to dry biomass coefficients for all methanogens are listed in Table 1. Cultures of *M. igneus* exhibited biomass aggregates and results did not show good linear regression in comparison to the other methanogens (Fig. S5). Investigating the pH at elevated headspace pressure revealed significant differences between different temperatures. At 65 °C the pH stabilizes to around 6, while at RT the pH drops to approximately 4.6 (Fig. S6). During cultivation of the thermophilic and hyperthermophilic methanogens a decrease in headspace pressure was visible right after of the cultivation, accelerating over time, indicating instant metabolic activity and growth of the microorganisms (Fig. S7-S10). This resulted in a steady decrease in cultivation time and shortening of the cycles, paralleled by an increase in the turnover rate until an inflection point is reached (Fig. 1), which is reflected in the peak of MER before gradually decreasing (Fig. 2). In the experiment with *M. marburgensis*, the decrease in metabolic activity additionally pinpoints the inflection point of the qCH_4_, but not of Y_CH4_, which was detected two cycles earlier (Fig. 3). The hyperthermophilic methanogens demonstrate different gas fermentation characteristics due to putative liquid limitations, whereby qCH_4_ reaches its maximum only during the initial one to two time points, while the inflection point, and simultaneous drop in MER, align more closely with the Y_CH4_ peak, revealing a relative push into the direction of energy conversion after catalytic inhibition occurred. Biomass production exceeded– except for *M. jannaschii*– albeit at a slower pace after the inflection point had been reached. *M. marburgensis*, *M. igneus* and *M. jannaschii* reached exponential growth with maximum growth rates of 0.55 ± 0.06 h^− 1^, 1.86 ± 0.10 h^− 1^, and 2.64 ± 0.10 h^− 1^ respectively, while *M. villosus* showed only linear behavior (1.03 ± 0.13 h^− 1^). This was also evidenced by the trends observed at the MER, where *M. villosus* displayed a different pattern, characterized by a slower initial increase but prolonged MER peak. *M. jannaschii* exhibited the highest MER of 58.24 ± 1.20 mmol L^− 1^ h^− 1^, followed by *M. igneus*, *M. marburgensis* and *M. villosus* with 54.07 ± 6.63 mmol L^− 1^ h^− 1^, 37.61 ± 3.37 mmol L^− 1^ h^− 1^ and 26.37 ± 0.61 mmol L^− 1^ h^− 1^ with *M. villosus*, respectively. The hyperthermophilic methanogens reach similar maxima in their biomass production, while *M. marburgensis* displayed the highest observed OD_578_ of 0.8223 ± 0.0601. However, in terms of qCH_4_, the hyperthermophiles exceed *M. marburgensis* with rates of 420.21 ± 33.39 mmol g^− 1^ h^− 1^ for *M. igneus*, 364.52 ± 25.50 mmol g^− 1^ h^− 1^ for *M. jannschii*, and 356.38 ± 20.79 mmol g^− 1^ h^− 1^ for *M. villosus*. After reaching the highest value at the start of the experiments, a decrease is observed shortly thereafter. This contrasts with *M. marburgensis*, where a more stable qCH_4_ is achieved (max at 169.59 ± 12.52 mmol g^− 1^ h^− 1^) over a longer period until the inflection point is reached. For an overview of the maximum values, please refer to Table 2. Considering the CH_4_ produced in moles also reveals either an exponential or linear progression (Fig. S11). Methanation by *M. maripaludis* was detected after a lag phase of approximately 10 h (Fig. 4, Fig. S12, Fig. S13) and showed exponential increase until an inflection point is reached. A decline in MER is apparent afterwards until the end of the methanation cycle leads to re-gassing of the cultivation bottles (Fig. 4, Fig. S13). MER_max_ was typically observed during the second cycle, followed by a mostly linear decrease until the headspace volume was replenished. The maximum MER drops with subsequent gassing cycles, while the duration of these cycles progressively increases.

**Table 2:** Measured physiological and biotechnological characteristics of the thermophilic/hyperthermophilic methanogens.

	OD_578_	x / g L^− 1^	µ / h^− 1^	MER / mmol L^− 1^ h^− 1^	qCH_4_ / mmol g^− 1^ h^− 1^	Y_CH4_ / g mmol^− 1^
*M. marburgensis*	0.8223 ± 0.0601	0.32 ± 0.02	0.55 ± 0.06	32.99 ± 0.13	169.59 ± 12.52	0.0030 ± 0.0004
*M. igneus*	0.4781 ± 0.0024	0.20 ± 0.00	1.86 ± 0.10	54.07 ± 6.63	420.21 ± 89.46	0.0060 ± 0.0008
*M. villosus*	0.5190 ± 0.0031	0.17 ± 0.00	1.04 ± 0.13	26.37 ± 0.61	356.38 ± 20.79	0.0038 ± 0.0009
*M. jannaschii* ^1^	0.4526 ± 0.0153	0.29 ± 0.01	2.64 ± 0.10	58.24 ± 1.20	364.52 ± 25.50	0.0090 ± 0.0013

Maximum detected values are displayed. Given values for Y_CH4_ / g mmol^− 1^ represent data at the inflection point. All experiments are *n* = 3. ^1^ Two OD measurements missing

## Discussion

### Set-up constraints of the GPC

The quantification of CH_4_ production kinetics is based on its partial pressure and can be calculated from the pressure kinetics apparent during methanation (Taubner and Rittmann 2016). Ensuring the gas tightness of the system is therefore of utmost importance and regular testing for the closure of the system is highly advised. Inaccuracy may arise through additional pressure loss, facilitated by damage to the rubber stopper. Excessive shaking can subject the needle to extreme stress, potentially causing breakage or wear of the rubber stopper. To mitigate possible risks, only new rubber stoppers were used. Due to the high cultivation temperature and the high re-gassing rate in some experiments, water vapor may form and accumulate within the pipes during the experiment. To prevent any liquids from entering the valves or the manometer, it is crucial to design the length of the tubes in a manner that allows condensation of water vapor to occur within the tubes. An attached sterile filter upstream of the cultivation bottle led to clocking due to vapor formation and deterred gas stream and pressure monitoring. However, sterile filters were installed during cultivation under mesophilic conditions, due to longer tubing and lower temperatures. To further facilitate sterile conditions, the tubing can be removed and autoclaved prior to conducting the experiments. In order to achieve an automated operation that ensures safe release and re-pressurizing of cultivation bottles, unwanted contact between the needle and liquid has to be avoided. This can occur frequently during heavy shaking and significantly increases the risk of culture being drawn into the system, leading to subsequent contamination and potential damage. A significant improvement would be the combination with a non-invasive OD sensor, which would eliminate processing time when measuring biomass concentrations.

### Data evaluation and uncertainty analysis

The application of several data filtering steps was successful in eliminating background data, present during the pressurizing phase at the beginning of each cycle. Due to the dissolution of gases into the liquid phase, a strong aberrant pressure loss occurs at the beginning of the cultivation cycles, which must be mitigated, due to an exaggerated CH_4_ production visible as sharp spikes in the MER plots. More rigorous data trimming results in greater data loss and must therefore be adjusted individually based on the organism and the experimental goals. However, preserving the complete cultivation duration is of minor importance when comparing physiological parameters. The methanation kinetics and biomass production kinetics must be evaluated separately, as the actual cultivation time differs from the cycles determined by the algorithm due to data trimming and data processing. Therefore, the CH_4_ production kinetics and the timepoint of biomass monitoring do not align accurately. The time intervals for calculating the growth rate were determined manually, based on the assumption that no significant metabolic activity occurs during pause of the GPC and the subsequent shutdown of the shaker. This is evident from the short pressure interval right before shaking and the GPC begins monitoring the headspace pressure. This pressure remains stable when cultivating *M. marburgensis*, while most cycles of the hyperthermophiles show an increase, presumably because the process temperature has not yet been reached. However, shaking rapidly induces biomethanation and biomass production suggesting that the discussed time interval can be neglected.

### Comparison of growth and CH_4_ production kinetics

Four different autotrophic, hydrogenotrophic methanogens were chosen and are among the highest performing CH_4_ producers so far identified (Mauerhofer et al. 2021). *M. marburgensis and M. jannaschii* additionally exhibits a broad range of fed-batch or continuous culture cultivations data (Mukhopadhyay et al. 1999; Seifert et al. 2014) or possess genetic tools (Susanti et al. 2019; Fink et al. 2021), while biotechnology and physiological studies concerning *M. igneus* and *M. villosus* are scarce.

Many gas fermentation bioprocesses are inhibited by either liquid or gas limiting conditions, a situation particularly present during closed batch cultivations. To improve GTR into the liquid phase an enlargement of the gas-liquid boundary is required, frequently achieved by rigorous stirring or shaking, or by increasing the driving force through applying barophilic conditions (Seifert et al. 2014; Takors et al. 2018; Pappenreiter et al. 2019; Mauerhofer et al. 2021). In order to push the metabolic activity of observed organism to the limits, even in closed batch cultivation mode, the unidirectional shaking was set to maximum while the GPC provides excess of gaseous substrate. During the experiments, no gas limitation was observed, as indicated in the acceleration of the conversion rate until an inflection point, defined as the timepoint where maximum conversion (k_min_ / bar h^− 1^) and MER_max_ occurs, is reached (Pappenreiter et al. 2019). It was not possible to measure the k_L_a using established methods, as the gassing in and gassing out method with O_2_ is only applicable in fed-batch fermentation with the appropriate probe, preferable with a short response time. Although probes for measuring H₂, CO₂, or CH₄ are available, they are not commonly used in closed batch systems.

The k_min_ determined for all experiments were around two to four times (2.8 to 4.1 bar h^− 1^) higher than the previously inspected values at 10 bar (max 1.52 ± 0.27 bar h^− 1^) and challenging most maximum conversion rates at 50 bars (0.85 ± 0.64 to 5.11 ± 0.55 bar h^− 1^) (Mauerhofer et al. 2021). It should be noted that our system allows for GTRs that are comparable to, or higher than those achieved in high-pressure experiments. However, making a precise statement about physiological properties was not possible, as the biomass was omitted during the analysis.

The inflection point, signaling the peak in MER, marks the precise moment when a putative liquid limitation occurs and, except for *M. igneus*, where one inflection point is off by one cycle, remains consistent within the experimental group. *M. marburgensis* shows optimal performance for about 7.06 h, in contrast to hyperthermophilic methanogens that reach the inflection point earlier, after 3 h for *M. igneus*, 3.3 h for *M. villosus*, and 3.7 h for *M. jannaschii*. Additionally, *M. igneus* and *M. jannaschii* show a sudden decrease in MER after reaching liquid limitation. Catalytic inhibition in *M. marburgensis* arises later, coinciding with a stable qCH_4_ up to that point. The hyperthermophilic methanogens decrease in qCH_4_ highlight early limitation right from the start of the cultivation and review different degrees of optimization potential of cultivation media and show specific differences between thermophilic and hyperthermophilic organisms. While the media of *M. marburgensis* was intensely optimized regarding trace elements (Schönheit et al. 1980; Abdel Azim et al. 2018), the hyperthermophilic 282c media was mostly unchanged or had been substituted with yeast extract or peptone for heterotrophic growth (Topçuoğlu et al. 2019), therefore compulsory inhibition of methanogenesis arise early. However, a thorough analysis of the limiting factors requires investigating the specific media requirements and conducting experimental testing of methanation under conditions of limitation or saturation. Previous studies highlight the correlation between trace elements on biomethanation efficiency (Abdel Azim et al. 2017; Rittmann et al. 2018). A limitation in trace element is therefore probable, deriving from lower absolute amounts and potentially higher demands and specificity, but might only explain the limitation localized by the inflection point. Additionally, the biological availability of chloride versus sulfate-based media needs to be verified. The necessity of Selenium (Se) for growth of methanogens was also reported, due to Se being part of enzymes responsible for energy metabolism (Grahame and Stadtman 1993). It can be assumed that selenite concentration is underrepresented, as higher concentrations were shown to be beneficial (Mukhopadhyay et al. 1999). The trends of qCH_4_ could also be a consequence of carbon regulation. The experiments indicate that during the initial phase of cultivation, the carbon flux shifts toward biomass production and shows a reversed trend after reaching the inflection point (Fig. 3). Therefore, the qCH_4_ decreases as the carbon flux in the biomass increases. This goes along with the theory that during sufficient nutrient supply, biomass growth is increased while the metabolism is less efficient. Conversely, under substrate limitation, growth decreases, but metabolic activity increases (Molenaar et al. 2009). This effect is more pronounced during hyperthermophilic growth, as during cultivation of *M. marburgensis*, where Y_CH4_ at the inflection point correlates with qCH_4max_, both exhibiting a positive trend that contradicts the proposed carbon flow regulation. Another possible explanation could be the instability of hyperthermophilic growth, where the rapid turnover leads to quick cell division and lysis, resulting in fewer enzymatically active cells overall. Furthermore, the pH was not measured during the experiment, as measuring pH in a closed environment is a highly inversive process. This is a known problem during closed batch gas fermentations and is extra challenging in anaerobic environments. An investigation of pH values under elevated pressures illustrates the decrease in CO_2_ solubility under elevated temperatures. It can therefore be assumed that the influence of the pressure as well as the CO_2_ solubility and consumption on the pH is limited under thermophilic and hyperthermophilic conditions.

The highest observed specific growth rate of *M. marburgensis* (µ_max_ = 0.55 ± 0.06 h^− 1^) was lower than the previously proposed (Schönheit et al. 1980) and confirmed (Abdel Azim et al. 2017) optimum of 0.69 h^− 1^. However, earlier experiments were carried out under optimized conditions in bioreactors (Abdel Azim et al. 2017). The qCH_4max_ of 169.59 ± 12.52 mmol g^− 1^ h^− 1^ is, under consideration of the standard deviation, almost equal to the maximum reported qCH_4_ (Abdel Azim et al. 2017), which is another indicator that the system during that time is not limited by the gas transfer or by liquid media depletion but by the physiology of the organism itself. Ascertained qCH_4max_ from *M. igneus*, *M. jannaschii*, *M. villous* were the highest ever recorded of any methanogen only challenged by Peillex et al. (Peillex et al. 1990). Here, reported qCH_4_ values exceed other published data by several factors, along with significant variations, potentially not reflecting actual physiological limits of *M. marburgensis* (Rittmann et al. 2018). The newly determined specific growth rates of the hyperthermophilic methanogens were, to our research, additionally the highest that had ever been reported (Miller et al. 1988; Mukhopadhyay et al. 1999; Topçuoğlu et al. 2019; Mauerhofer et al. 2021). Investigating qCH_4_, MER, µ and Y_CH4_ shows lack of homogeneity in some cycles. Therefore, the statistical analysis was not conducted with the complete data set. Statistical relevance was found in the first cycle of qCH_4_ between *M. marburgensis* and the hyperthermophiles but not between the hyperthermophilic organisms themselves. This highlights the methanation capacities between organisms of different temperature regimes. The biomethanation capacities of tested hyperthermophiles are probably too closely related to detect meaningful differences.

### Long term cultivation with ***M. maripaludis***

*M. maripaludis* is one of the best studied autotrophic, hydrogenotrophic methanogens and exhibit an advanced molecular toolkit, including CRISPR-mediated genome editing, that labels the organism as a valuable cell factory well-suited for physiological studies and the production of a wide range of different products (Walters et al. 2011; Richards et al. 2016; Bao et al. 2022; Li et al. 2022; Xu et al. 2023; Du et al. 2024). As a result, increased efforts have been made towards improving biotechnological capability, such as in the production of biomass (Palabikyan et al. 2022).

*M. maripaludis* was successfully cultivated over 80 h while constant monitoring and adjusting of the headspace pressure. Figure 4 and Fig. S13 delineate the gas consumption during cultivation with suboptimal culture to headspace volume ratio. While the exponential increase in MER after the lag phase suggests exponential growth behavior, an inflection point is reached soon after, signaling the transfer from gas unlimited to limited conditions. Afterward, a decreasing trend in the MER, starting anew with each gassing cycle, can be observed. It is assumed that during gas limited conditions and biomass saturation the MER matches the GTR. Since the GTR is the product of the k_L_a and the driving force (Δc) from the gas to the liquid phase, and a stable k_L_a is assumed during the cultivation process, the MER can be considered a direct function of the pressure. However, the total CH_4_ production decreases with each cycle, accompanied by an increase in cultivation time, evident by the reduced slope and the transition to more curved methanation kinetics over time. This hypothesis requires further testing, as cultivation results obtained from *M. maripaludis* using CO_2_ as carbon source reveal liquid limitations as early as the second cycle. This is evidenced by a decline in MER at consistent pressure levels across successive methanation cycles (Fig. S14), a trend also apparent in the wavelike pattern of the total amount of produced CH_4_ in mol (Fig. S15).

In conclusion: The GPC facilitates automated gassing of a wide range of different cultivation bottles in closed batch settings and allows for monitoring and regulation of the headspace gas pressure in real time over virtually unlimited duration. Here, the GPC has been specifically used to elucidate physiological and biotechnological characteristics of autotrophic, hydrogenotrophic methanogens. Further testing is needed to adapt the system for other gas conversion processes, such as those that support microbial activity followed by a fixed stoichiometry. First, the GPC allows quantification of physiological limits of autotrophic, hydrogenotrophic methanogens by eliminating gas limitation during growth of organisms and facilitates near optimal cultivation conditions in closed batch setups. As a result, previously unknown and very high qCH_4_ values for three hyperthermophilic methanogens have been determined. Second, the device can accurately determine the occurrence of liquid limitations by visualizing the conversion point of metabolic inhibition. This allows for conclusions about limiting components and can be further exploited by high-throughput experiments. Third, the GPC may act as a tool for generating biomass on a large scale and by reaching specific growth rates that would not be possible in established manual closed batch cultivation settings. Finally, the GPC demonstrates application flexibility towards various applications in the fields of gas fermentation with hydrogenotrophic methanogens and archaea biotechnology.

## Electronic supplementary material

Below is the link to the electronic supplementary material.

Supplementary Material 1

## Data Availability

The datasets generated and/or analysed during the current study are available in the PHAIDRA repository of Universität Wien, 10.25365/phaidra.518; 10.25365/phaidra.519; 10.25365/phaidra.597; 10.25365/phaidra.598.
