# Supplementary material for: Method for automated high performance closed batch cultivation of gas-utilizing methanogens
Source: AMB Express. 2025 Apr 29;15:67. doi: 10.1186/s13568-025-01872-y (PMC12040808; doi:10.1186/s13568-025-01872-y)
Supplement: Supplementary file 1 — Supplementary Material 1 [file 13568_2025_1872_MOESM1_ESM.pdf]

Supplementary material to: Method for automated high performance closed batch cultivation of gas-fermenting methanogens

Walter Hofmann[1,2,#], Marco Orthofer[3,#], Nicolás Salas Wallach[1,2], Aquilla Ruddyard[1], Markus Ungerank[4], Christian Paulik[3], Simon K.-M. R. Rittmann[1,*]

[1]Archaea Physiology & Biotechnology Group, Department of Functional and Evolutionary Ecology, Universität Wien, Wien, Austria
[2]Acib GmbH, Wien, Austria
[3]Institute for Chemical Technology of Organic Materials, Johannes Kepler Universität Linz, Linz, Austria
[4]Creonia e.U., Perg, Austria

[#]These authors contributed equally to this work

[*]Corresponding author:
Dr. Simon K.-M. R. Rittmann, Privatdoz.
Archaea Physiology & Biotechnology Group
Department of Functional and Evolutionary Ecology
Djerassiplatz 1
Universität Wien
1030 Wien
Austria
Email: simon.rittmann@univie.ac.at

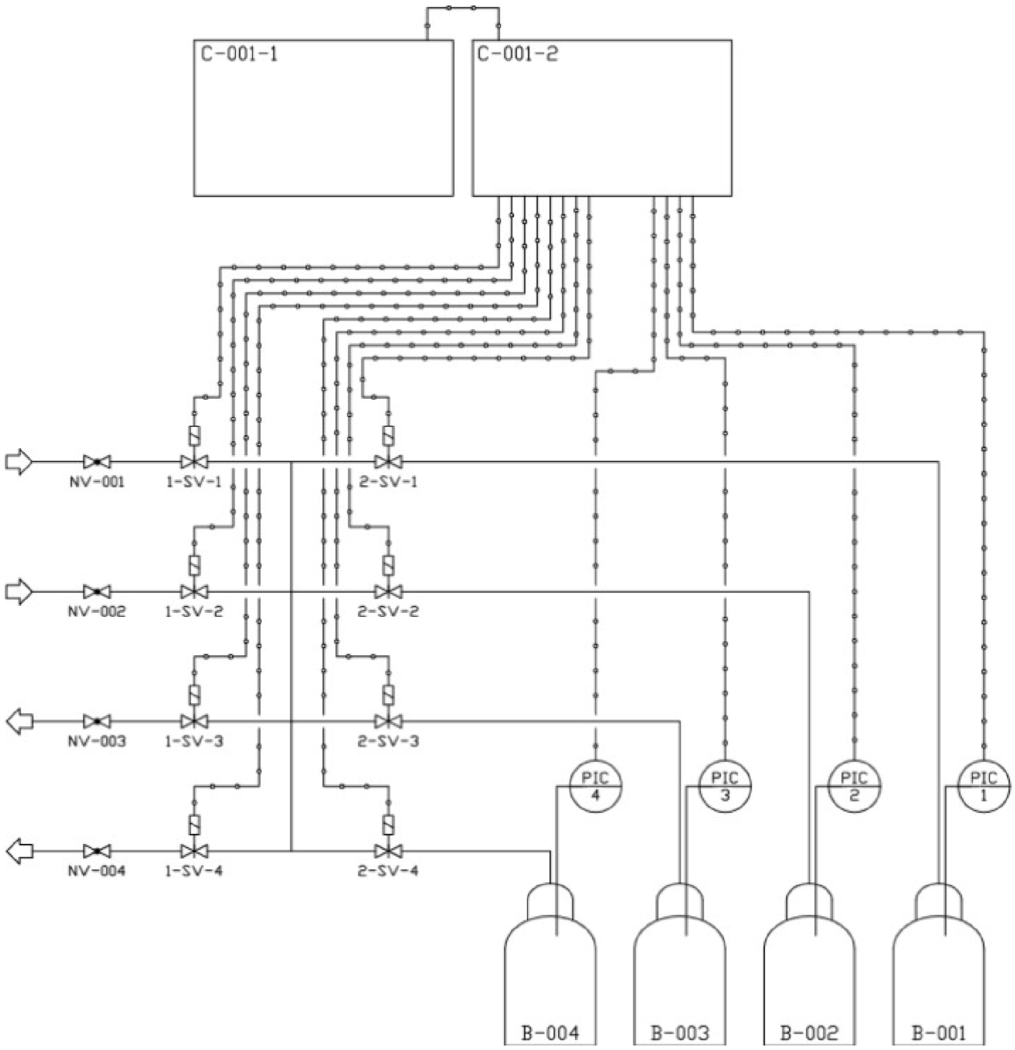

Fig. S1: Instrumentation diagram of the GPC. One unit consists of eight valves (SV1-8) equipped with four pressure sensors (PIC1-4) and four throttling valves (NV1-4) connected to a controlling device allowing for pressure monitoring of four cultivation bottles simultaneously. Individual components are connected via pressure resistant tubing.

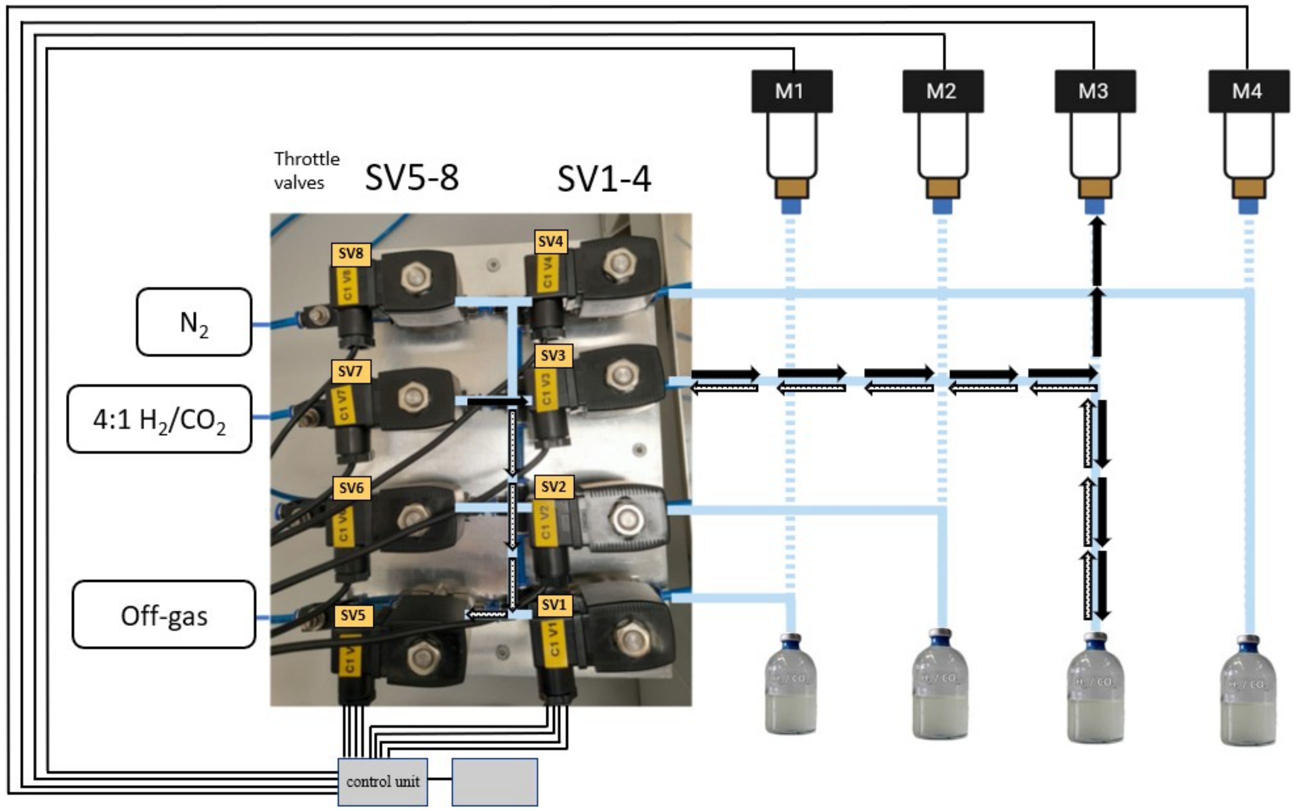

Fig. S2: Technical setup of the GPC and illustration of its functionality. Eight valves (SV1–SV8) are arranged in two parallel rows and connected to a control device along with four pressure sensors (M1–M4) and four throttling valves. All components are interconnected using pressure-resistant tubing. Valves 7 and 9 are opened simultaneously, allowing the gas (H_2/CO_2) to stream into the serum bottles, resulting in a pressure buildup (black arrows). During sparging, Valves 5 and 8 are opened, reducing the pressure in the serum bottles (white arrows). The flushed gas is safely discarded through the fume hood (Sterile filters not shown).

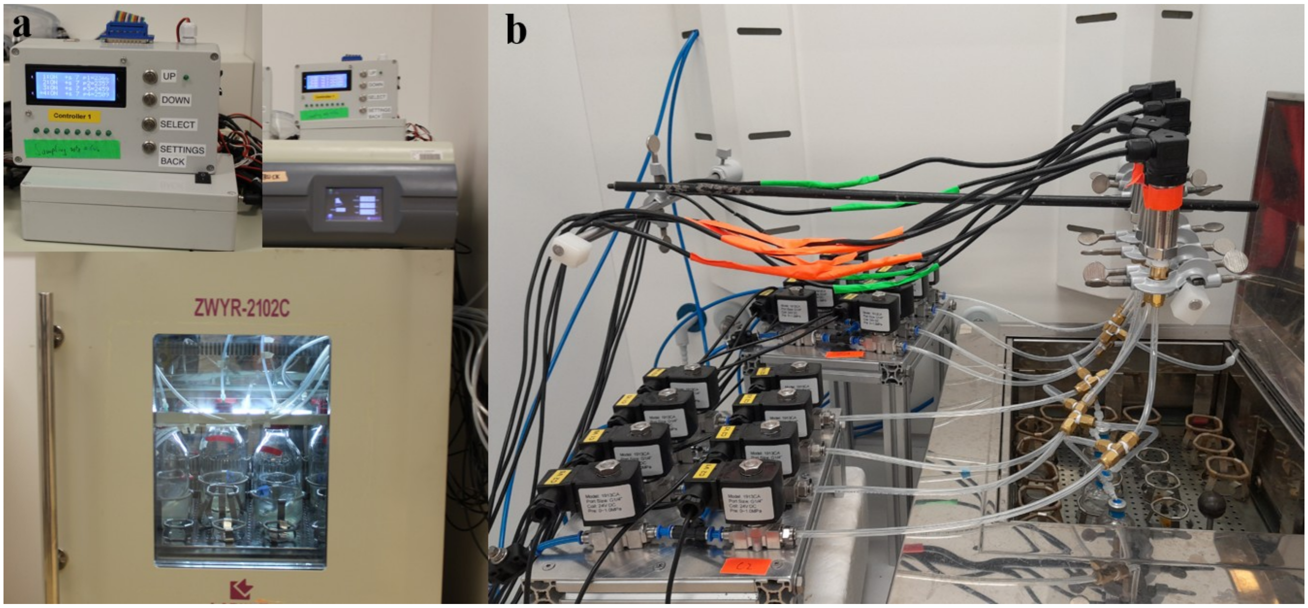

Fig. S3: Images of the experimental setup of the GPC. a. The gas lines are introduced into the air incubator from the side and attached to the bottles. The controlling system for data monitoring and power supply is additionally shown. b. GPC set-up in the fume hood.

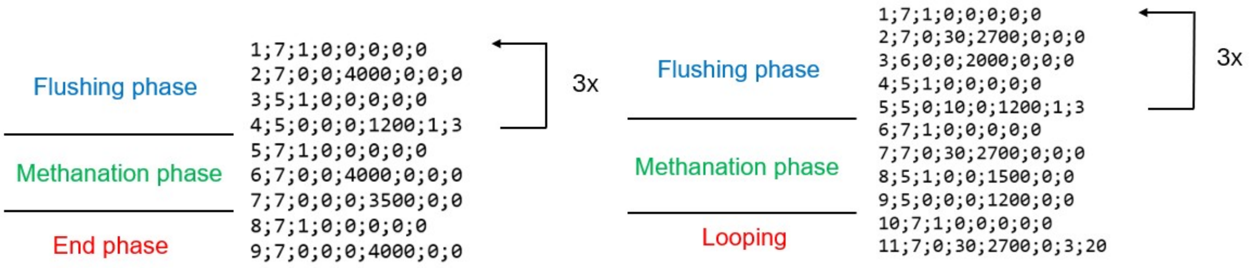

Fig. S4: Representation of the programs used in CSV format. Left: Program used for cultivation of thermophilic/hyperthermophilic methanogens. After the end phase, one methanation cycle concludes and OD_{578} is measured. Right: Program applied for autonomous overnight cultivation with *Methanococcus maripaludis*. After the methanation phase the culture is repressurized and looped back to the flushing phase (20x). Prior to operation, the required program must be defined and comprise of a series of executable steps. Column 1 (Step): Program step with consecutive number (1-30); Column 2 (valve): Number of the valve to be opened or closed (5,6,7 or 8) (Note: Valves 1 to 4 are automatically controlled); Column 3 (statusValve): 0 for closed valve, 1 opens the corresponding valve; Column 4 (ValveOnTime): Indicates the maximum opening time of the valve in seconds; Column 5 (PressureHigher): The step ends when the detected pressure in mbar is higher than the set pressure value; Column 6 (PressureLower): The step ends when the measured pressure in mbar is lower than the set pressure value; Column 7 (Jump): The program continues with the step specified after the criteria in columns 4, 5 or 6 is met; Column 8 (Number of jumps): Indicates the number of times the program should repeat the step specified in column 7. All valves can also be controlled manually.

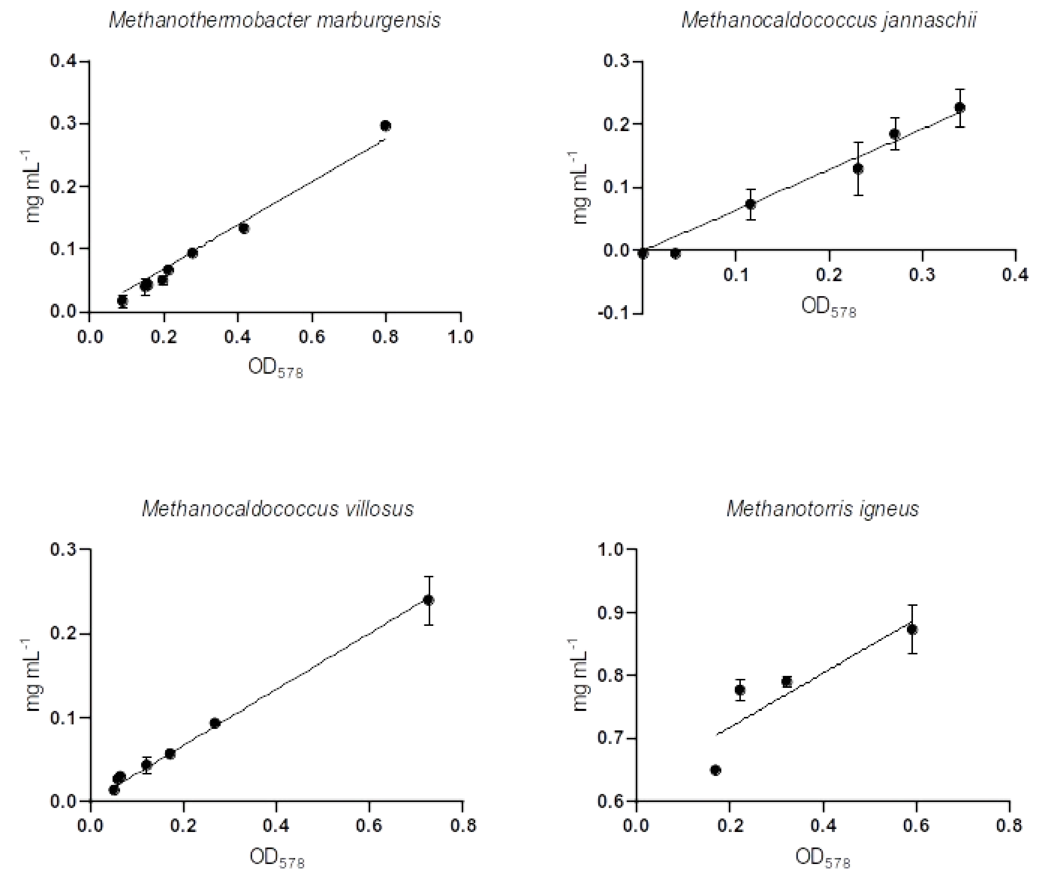

Fig. S5: Dry biomass in mg mL^{-1} blotted over OD_{578}. Linear regression forced through zero (except *M. igneus*). Slope determines the OD_{578} to dry biomass coefficient x.

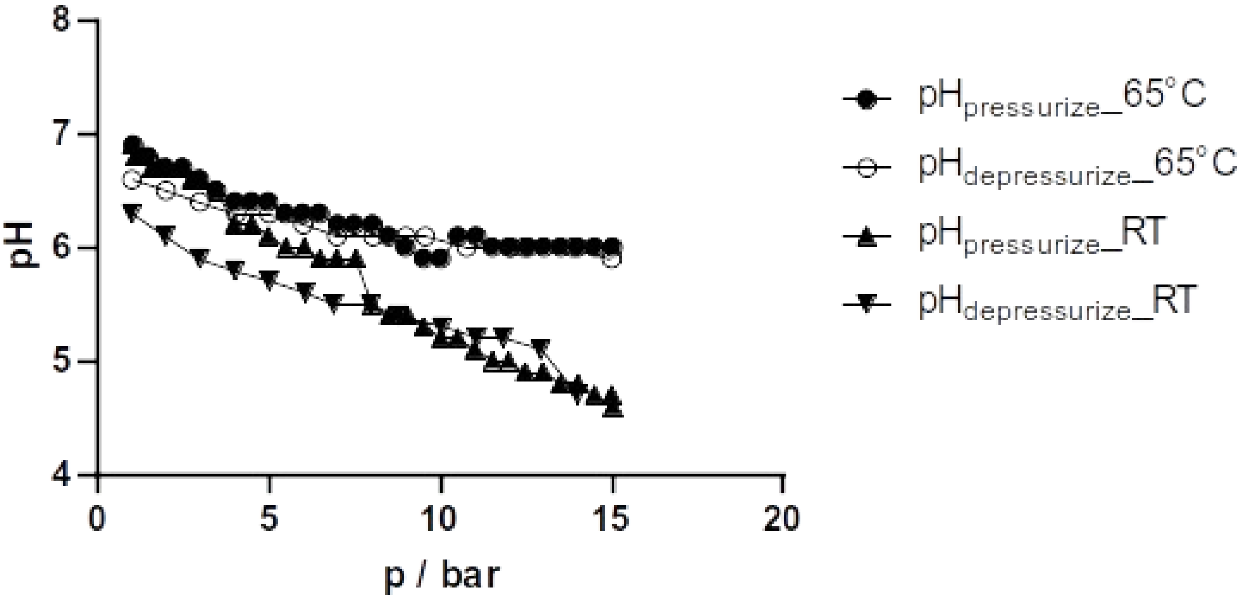

Fig. S6: The pH dependence on bar overpressure was analyzed using a 4:1 H_2/CO_2 gas mixture at room temperature (RT) and 65°C. pH measurements were taken during the pressurization and depressurization of the medium.

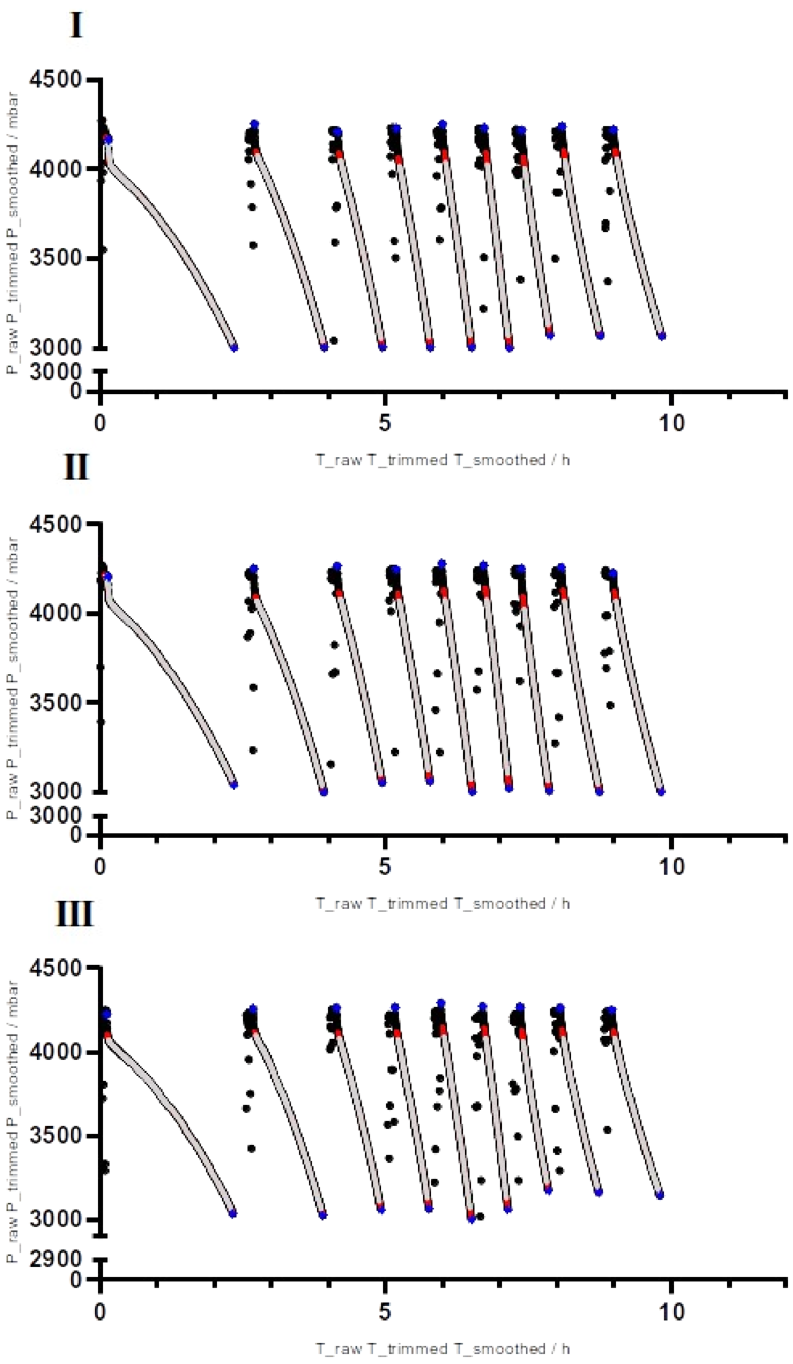

Fig. S7: Pressure kinetics (in bar overpressure) of the methanation experiments of *M. marburgensis* in triplicate design. Raw data (black) represents values extracted from the GPC. Trimmed data (red) are obtained after applying data filtering. The smoothed data set (grey) represents pressure kinetics after applying the smoothing factor and is used for subsequent calculation of the physiological parameters. Cultivation time used for calculation of μ / h^{-1} is highlighted in blue and represent start and beginning of a cultivation cycle (total 9).

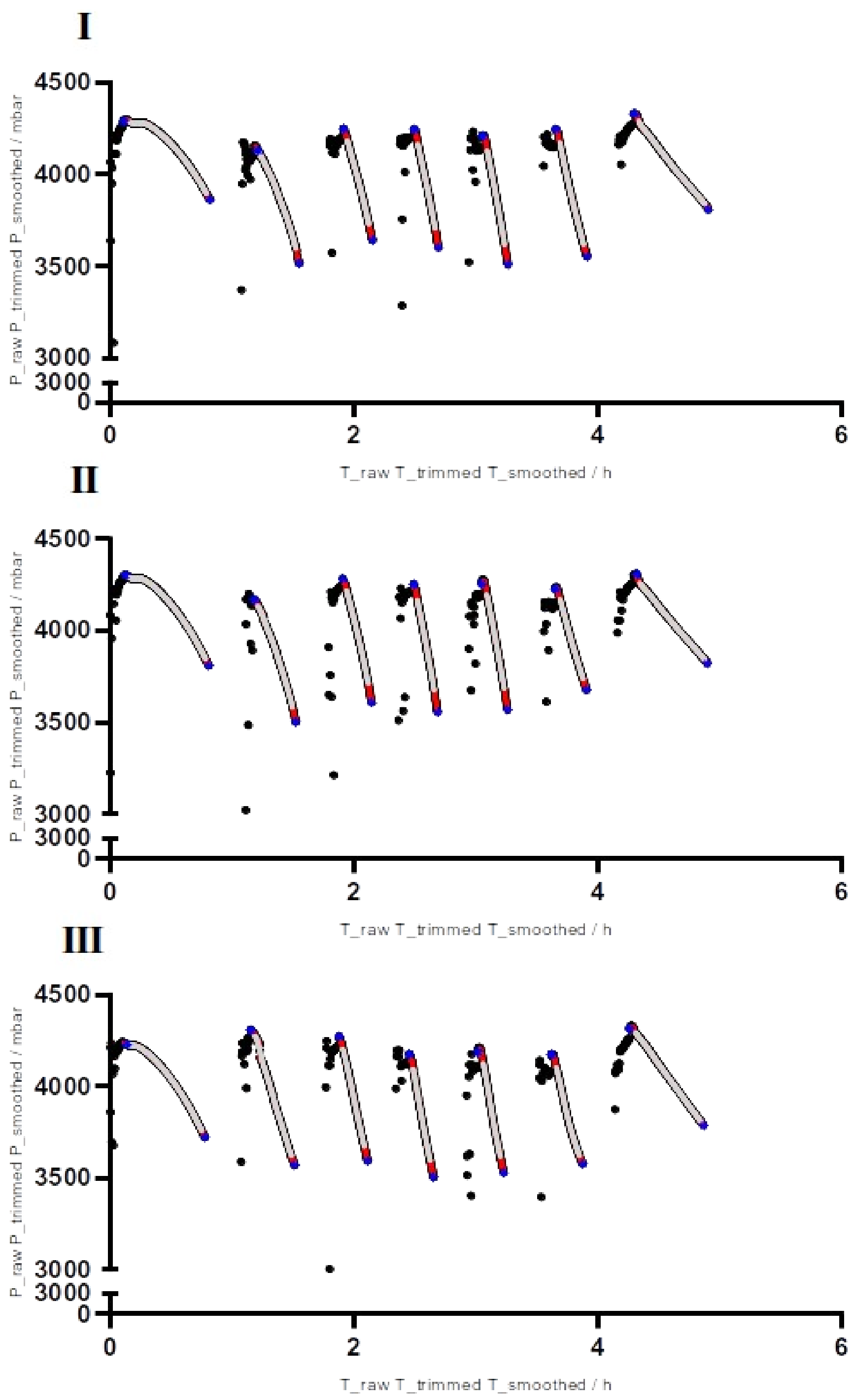

Fig. S8: Pressure kinetics (in bar overpressure) of the methanation experiments of *M. igneus* in triplicate design. Raw data (black) represents values extracted from the GPC. Trimmed data (red) are obtained after applying data filtering. The smoothed data set (grey) represents pressure kinetics after applying the smoothing factor and is used for subsequent calculation of the physiological parameters. Cultivation time used for calculation of μ / h^{-1} is highlighted in blue and represent start and beginning of a cultivation cycle (total 7).

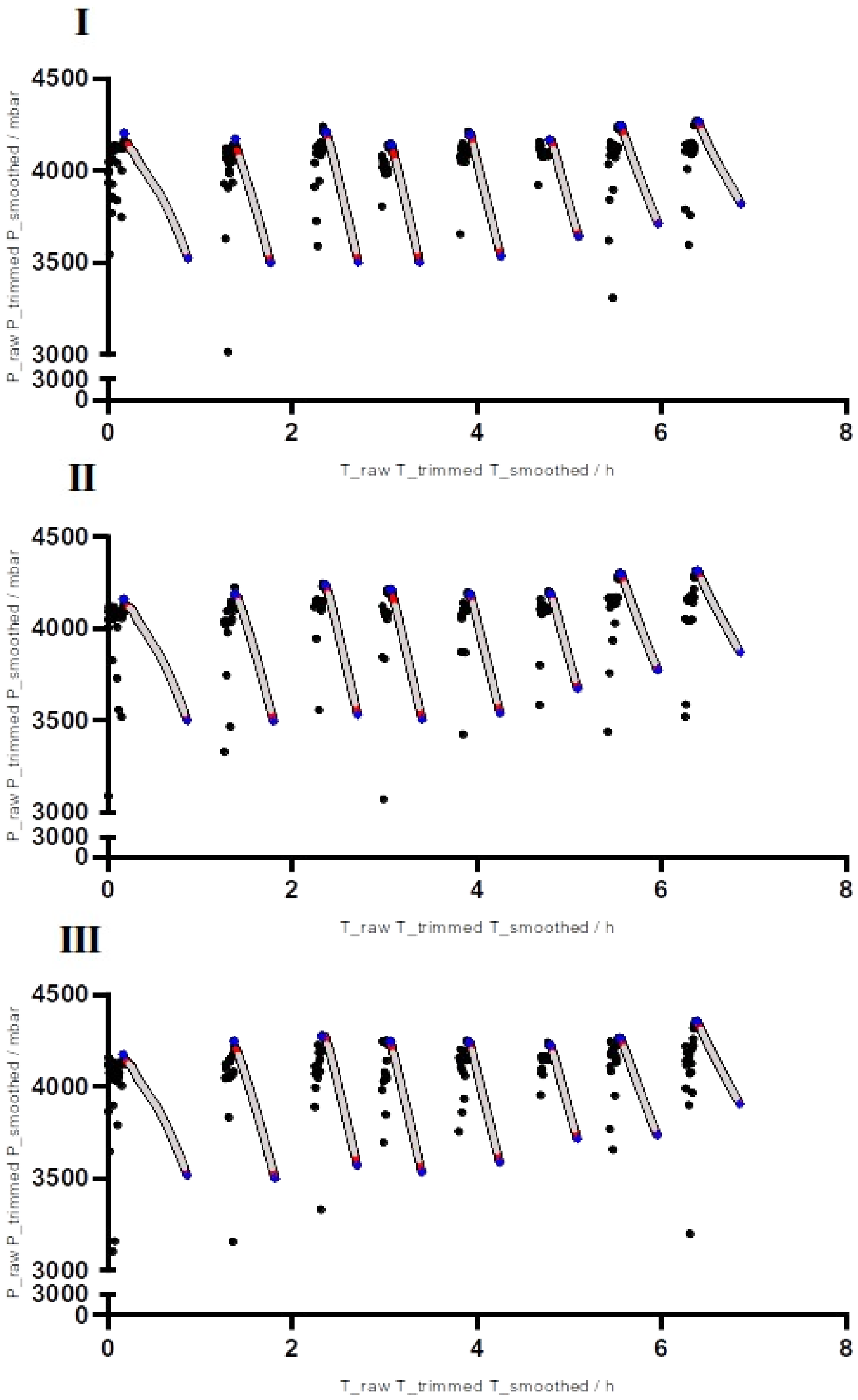

Fig. S9: Pressure kinetics (in bar overpressure) of the methanation experiments of *M. villosus* in triplicate design. Raw data (black) represents values extracted from the GPC. Trimmed data (red) are obtained after applying data filtering. The smoothed data set (grey) represents pressure kinetics after applying the smoothing factor and is used for subsequent calculation of the physiological parameters. Cultivation time used for calculation of μ / h^{-1} is highlighted in blue and represent start and beginning of a cultivation cycle (total 8).

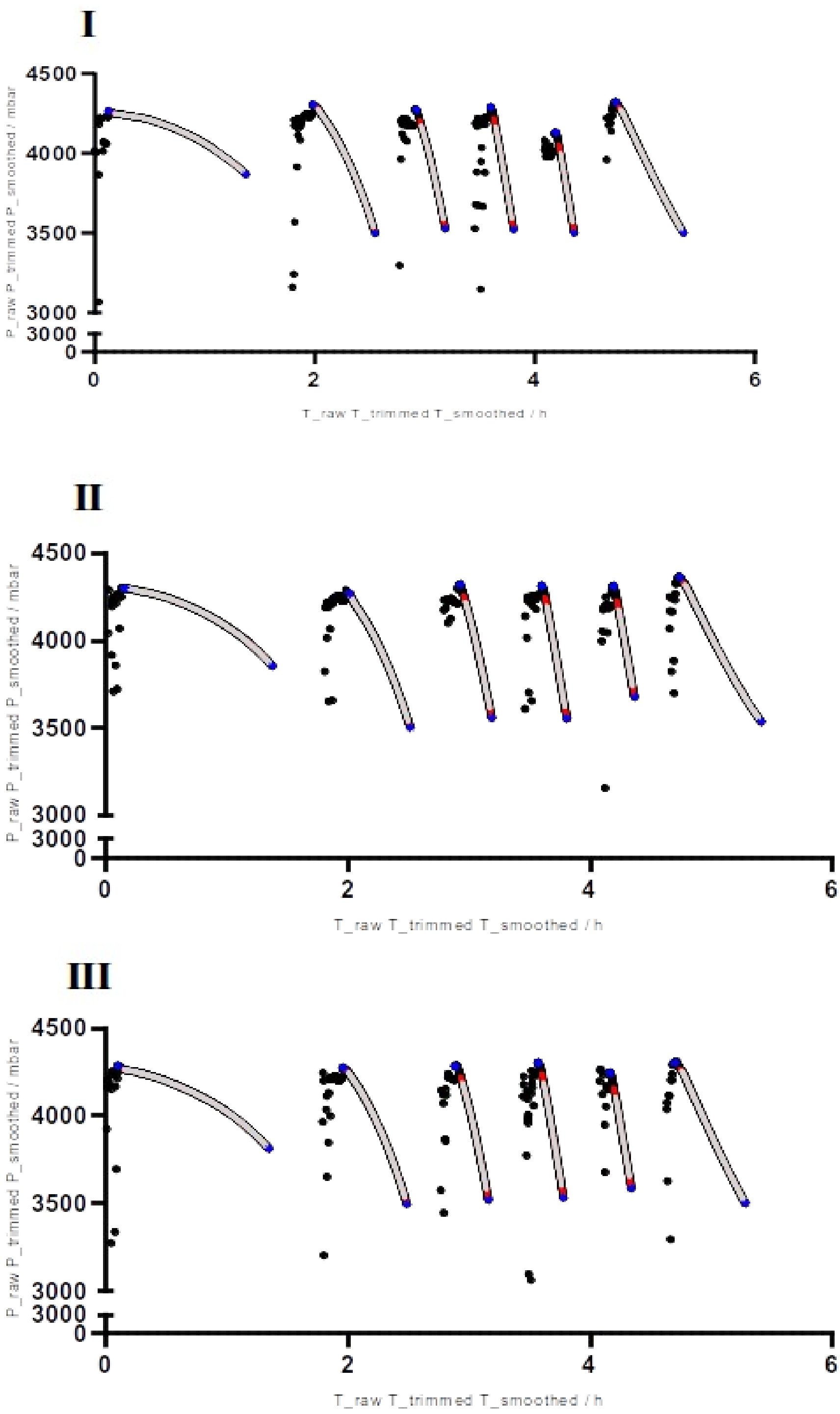

Fig. S10: Pressure kinetics (in bar overpressure) of the methanation experiments of *M. jannaschii* in triplicate design. Raw data (black) represents values extracted from the GPC. Trimmed data (red) are obtained after applying data filtering. The smoothed data set (grey) represents pressure kinetics after applying the smoothing factor and is used for subsequent calculation of the physiological parameters. Cultivation time used for calculation of μ / h^{-1} is highlighted in blue and represent start and beginning of a cultivation cycle (total 6).

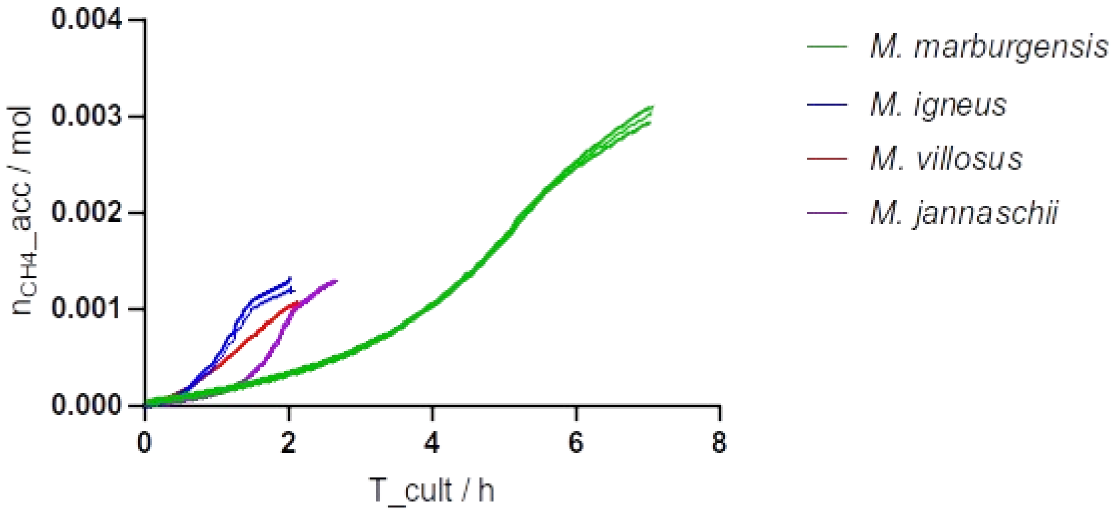

Fig. S11: Accumulated moles of CH_4 produced determined in real time over the cultivation time. All experiments were performed in triplicates. Standard deviations are indicated via parallel lines.

I

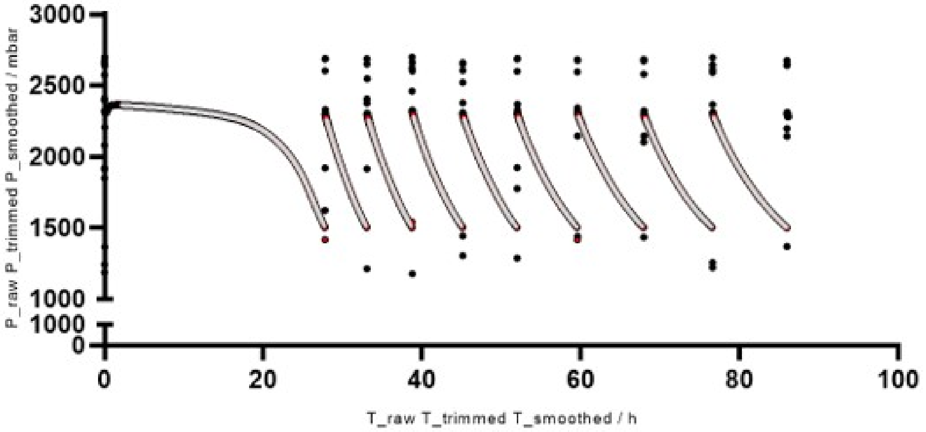

II

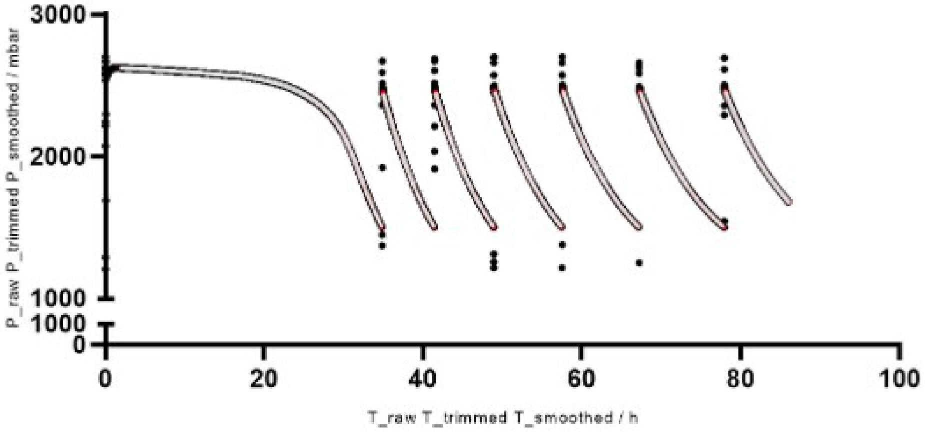

III

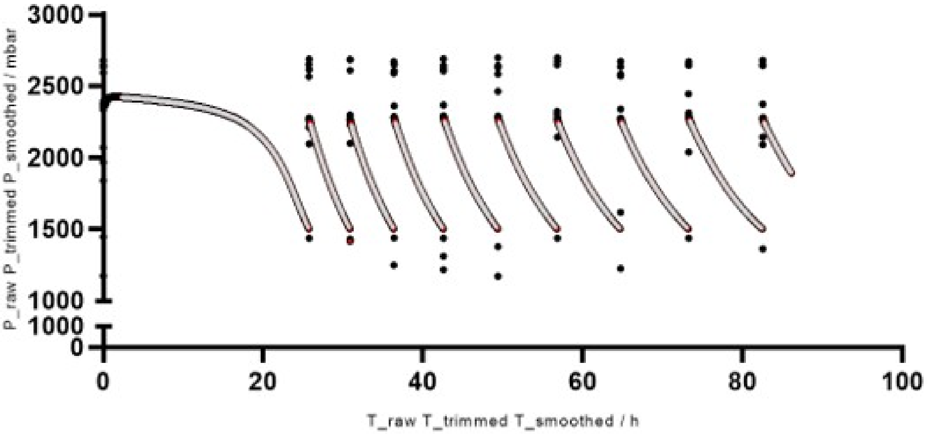

Fig. S12: Pressure kinetics (in bar overpressure) of the methanation experiments of *M. maripaludis* in triplicate design. Raw data (black) represents values extracted from the GPC. Trimmed data (red) are obtained after applying data filtering. The smoothed data set (grey) represents pressure kinetics after applying the smoothing factor and is used for subsequent calculation of the physiological parameters.

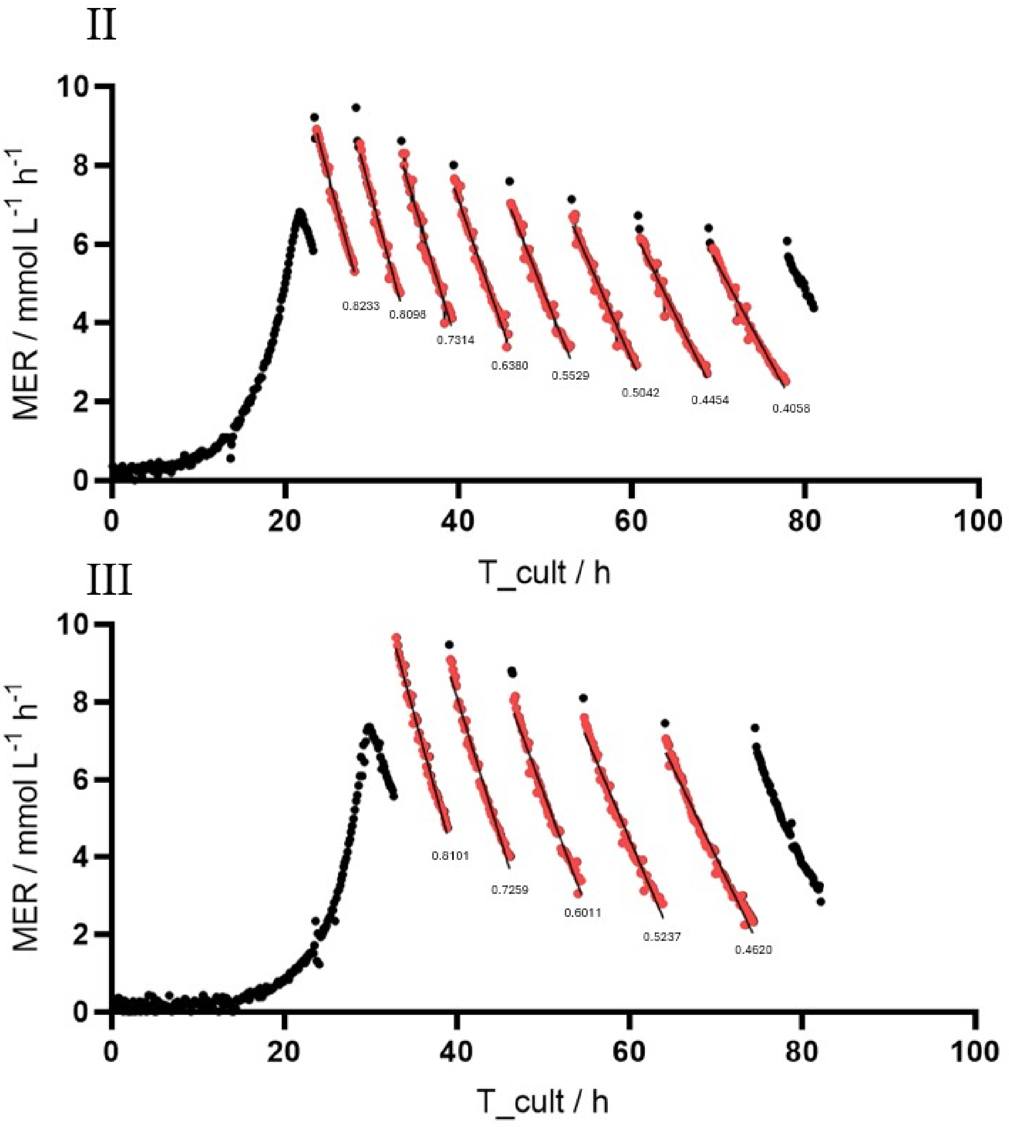

Fig. S13: Methanation kinetics of *M. maripaludis* cultivation. Experiments were conducted in triplicate, with a single experiment shown in Fig. 4. The slope of the linear regression was calculated for each cycle.

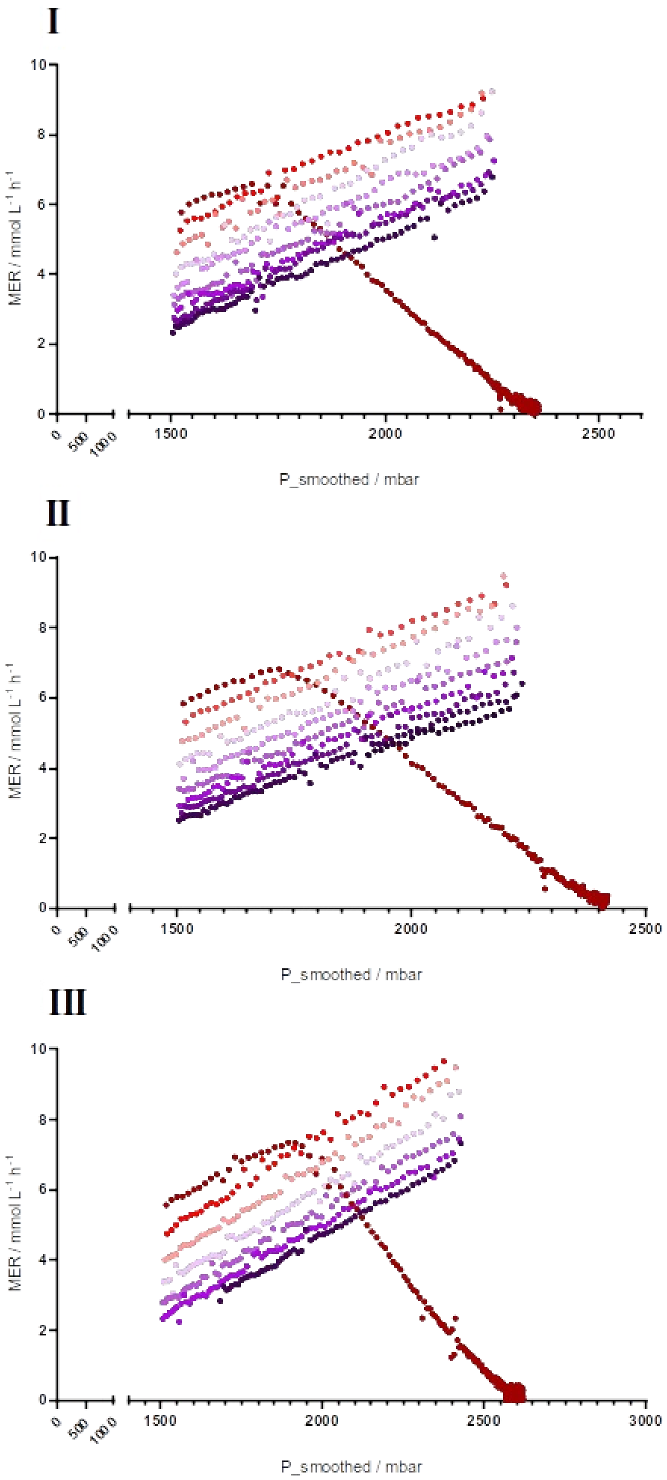

Fig. S14: The MER for *M. maripaludis* is plotted against the corresponding pressure. Individual gassing cycles are represented using a color gradient, ranging from dark red to purple.

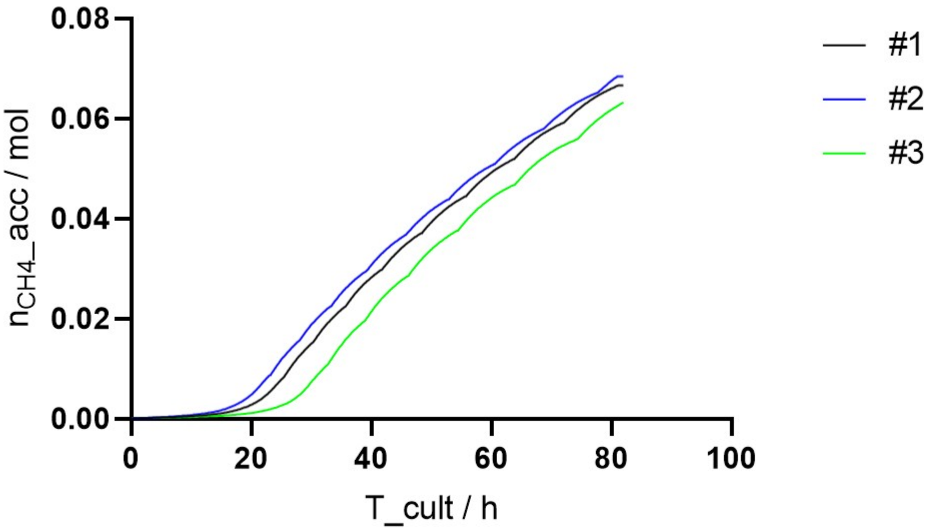

Fig. S15: Accumulated moles of CH_4 produced determined in real time over the cultivation time for *M. maripaludis*. All experiments were performed in triplicates. Individual experiments are shown.
